# Beyond geometry orders: uncovering bonding-heterogeneity-dominated structure-relaxation coupling in glasses

**DOI:** 10.1093/nsr/nwag006

**Published:** 2026-01-15

**Authors:** Liang Gao, Jia-Qi Gao, Qing-Zhou Bu, Qun Yang, Yang Sun, Kai-Ming Ho, Qi Wang, Jeppe C Dyre, Hai-Bin Yu

**Affiliations:** Wuhan National High Magnetic Field Center and School of Physics, Huazhong University of Science and Technology, Wuhan 430074, China; Science and Technology on Surface Physics and Chemistry Laboratory, Mianyang 621908, China; Wuhan National High Magnetic Field Center and School of Physics, Huazhong University of Science and Technology, Wuhan 430074, China; Wuhan National High Magnetic Field Center and School of Physics, Huazhong University of Science and Technology, Wuhan 430074, China; Wuhan National High Magnetic Field Center and School of Physics, Huazhong University of Science and Technology, Wuhan 430074, China; College of Physics and Electronic Engineering, Chongqing Normal University, Chongqing 401331, China; Department of Physics, Xiamen University, Xiamen 361005, China; Department of Physics and Astronomy, Iowa State University, Ames, IA 50011, USA; Science and Technology on Surface Physics and Chemistry Laboratory, Mianyang 621908, China; Glass and Time, IMFUFA, Department of Science and Environment, Roskilde University, Roskilde DK-4000, Denmark; Wuhan National High Magnetic Field Center and School of Physics, Huazhong University of Science and Technology, Wuhan 430074, China

**Keywords:** relaxation dynamics, glass transition, metallic glass, chemical interaction

## Abstract

The *microstructure determines properties* paradigm applies well to crystalline materials but struggles with amorphous systems. While researchers have long sought to link amorphous structures to macroscopic properties, traditional analyses focus on geometric packing, which our study reveals to be insufficient. We demonstrate this using two Pd-based metallic glasses, $\rm {Pd}_{40}\rm {Cu}_{40}\rm {P}_{20}$ and $\rm {Pd}_{40}\rm {Ni}_{40}\rm {P}_{20}$, which exhibit nearly identical geometries but different secondary relaxations. Electronic structure analysis uncovers the key distinction: $\rm {Pd}_{40}\rm {Cu}_{40}\rm {P}_{20}$ has weaker Cu–P bonds and a less developed covalent network, enabling string-like atomic motions that drive pronounced relaxation, whereas $\rm {Pd}_{40}\rm {Ni}_{40}\rm {P}_{20}$’s stronger Ni–P interactions create a more constrained network. These findings highlight the critical role of electronic interactions and bonding fluctuations—beyond geometry—in governing glass dynamics. By integrating experiments and deep-learning simulations, we bridge the gap between local bonding heterogeneity and macroscopic behavior, offering new design principles for amorphous materials that prioritize electronic structure over purely geometric order. This advances glass physics by emphasizing the need to incorporate chemical interactions into structural analyses.

## Teaser

Why do amorphous materials behave differently despite similar structures? This study shows that electronic interactions, rather than geometry alone, play a decisive role in controlling glass dynamics and properties.

## INTRODUCTION

Glasses are an significant focus of contemporary basic research and technological development [1–18]. Although the composition of a glass obviously dictates its mechanical and other functional characteristics, even minor variations may lead to substantial differences in properties such as glass-forming ability (GFA), mechanical strength and magnetic behavior [19–24]. The composition affects both atomic and electronic structure [13,25], and fully determining the connection between composition, structure and dynamics remains a formidable task.

Currently, structural analysis of glassy materials focuses mainly on short- and medium-range geometries, including free volume, common-neighbor analysis, local five-fold symmetry and icosahedral orders [26–30], along with advanced methods such as cluster alignments, smooth overlap of atomic positions (SOAP) and inherent-structure minimal displacements [31–33]. While significant efforts have been made to link these features to atomic motions and global relaxation dynamics, their success varies [13]. A frequently neglected factor is the fluctuation of chemical interactions, or bonding heterogeneity. This may be less critical in simple models based on, for example, hard-sphere systems, but becomes crucial in real multi-element materials such as metallic glasses (MGs) [20,34–38].

Metallic glasses that incorporate metalloid elements such as phosphorus (P), sulfur (S), oxygen (O) and silicon (Si) cannot simply be modeled as hard-sphere systems due to their complex chemical interactions, and such systems often have intriguing properties [39–42]. A notable example is $\rm {Pd}_{40}\rm {Ni}_{40}\rm {P}_{20}$, which possesses the highest GFA among all known ternary metallic glasses [43]. Substituting Ni with Cu to create $\rm {Pd}_{40}\rm {Cu}_{40}\rm {P}_{20}$ dramatically reduces the GFA and simultaneously results in another extreme: the most pronounced secondary $\beta$-relaxation peaks observed to date in metallic glasses [20].

Why do seemingly minor compositional changes—such as substituting chemically and structurally similar elements—in some cases lead to pronounced differences in GFA and dynamics, e.g., in the $\beta$-relaxation behavior? Despite similar atomic sizes and comparable chemical affinities, such substitutions can lead to unexpected and sometimes drastic changes in dynamics and stability. This paradox underscores the complexity of metallic glasses, where a delicate balance between metallic, covalent and even ionic interactions often plays a critical role in governing structural and dynamic properties [13,44–47].

Density-functional-theory (DFT)-based simulations by Guan *et al.* [48] attribute the exceptional GFA of the $\rm {Pd}_{40}\rm {Ni}_{40}\rm {P}_{20}$ glass to its intriguing short-range order and localized electronic distributions. However, the limited model size (200 atoms) and relatively short simulation time of DFT simulations make it difficult to properly capture relaxation processes such as the $\beta$ relaxation, a point of focus below. Although classical force fields offer a route to simulate larger systems over longer time scales [33,36,49–55], the accuracy of these simulations may be compromised by such simplified potentials, and they often fail to replicate the experimental dynamics.

To date, no simulation frameworks have successfully reproduced the strikingly different relaxation dynamics observed for $\rm {Pd}_{40}\rm {Ni}_{40}\rm {P}_{20}$ and $\rm {Pd}_{40}\rm {Cu}_{40}\rm {P}_{20}$. We address this challenge below by utilizing a set of realistic PdNi(Cu)P deep potentials (DPs) under the DeepMD framework, trained on extensive DFT calculations [18,56]. This approach allows the scalability of traditional force fields to be merged with the precision of DFT [57–61], enabling the capture of subtle chemical differences that dictate the unique relaxation dynamics (compare Figs S1–S4).

## RESULTS AND DISCUSSION

**Figure 1.**
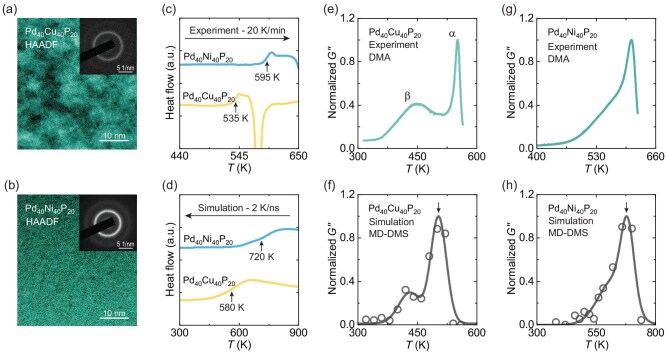
Significant differences in the thermal and dynamic mechanical properties of the two systems are established by experiments and simulations. (a and b) HAADF-STEM images (scale bars: 10 nm) of $\rm {Pd}_{40}\rm {Cu}_{40}\rm {P}_{20}$ and $\rm {Pd}_{40}\rm {Ni}_{40}\rm {P}_{20}$ glasses at room temperature. The insets show selected-area electron diffraction patterns, confirming the amorphous nature of the samples. (c) Differential scanning calorimetry heat-flow curves for as-cast $\rm {Pd}_{40}\rm {Cu}_{40}\rm {P}_{20}$ (yellow) and $\rm {Pd}_{40}\rm {Ni}_{40}\rm {P}_{20}$ (blue) glasses at a heating rate of 20 K/min. The glass-transition temperatures $T_g$ are 535 and 595 K, respectively. (d) Simulated heat-flow evolution during a continuous cooling at the rate of $R=2$ K/ns for $\rm {Pd}_{40}\rm {Cu}_{40}\rm {P}_{20}$ (yellow) and $\rm {Pd}_{40}\rm {Ni}_{40}\rm {P}_{20}$ (blue) glasses, with corresponding $T_g$ of 580 and 720 K, respectively. (e and g) Loss modulus obtained from experimental DMA for as-cast $\rm {Pd}_{40}\rm {Cu}_{40}\rm {P}_{20}$ and $\rm {Pd}_{40}\rm {Ni}_{40}\rm {P}_{20}$ glasses at 1 Hz. (f and h) Loss modulus from deep-potential-based DMS simulations with an oscillation period of 100 ns for $\rm {Pd}_{40}\rm {Cu}_{40}\rm {P}_{20}$ and $\rm {Pd}_{40}\rm {Ni}_{40}\rm {P}_{20}$ glasses. The corresponding $\alpha$-relaxation temperatures $T_{\alpha }$ are 510 and 675 K, respectively (marked by the arrows).

We begin by demonstrating that our PdNi(Cu)P DPs capture key relaxation characteristics of experiments. Panels a and b of Fig. 1 show high-angle annular dark-field scanning transmission electron microscopy (HAADF-STEM) images of $\rm {Pd}_{40}\rm {Cu}_{40}\rm {P}_{20}$ and $\rm {Pd}_{40}\rm {Ni}_{40}\rm {P}_{20}$ glasses at room temperature. The $\rm {Pd}_{40}\rm {Cu}_{40}\rm {P}_{20}$ glass features a visibly more heterogeneous microstructure at long range [62–65]. Figure 1c presents calorimetric data for the two glasses, showing that $\rm {Pd}_{40}\rm {Cu}_{40}\rm {P}_{20}$ exhibits a lower glass-transition temperature than $\rm {Pd}_{40}\rm {Ni}_{40}\rm {P}_{20}$. Further details of the experimental results can be found in Figs S5–S8.

Figure 1d shows the heat-flow evolution of the two model glasses during cooling simulations. These data are consistent with the experimental finding that the $\rm {Pd}_{40}\rm {Cu}_{40}\rm {P}_{20}$ glass transition takes place at a lower temperature (see also Fig. S8). Panels e, g and f, h of Fig. 1 respectively present relaxation spectra of the two glasses from experiments and simulations; additional relaxation spectra with different oscillation periods can be found in Figs S6 and S15. From experiments, the loss spectrum of $\rm {Pd}_{40}\rm {Cu}_{40}\rm {P}_{20}$ exhibits two distinct peaks: a high-temperature peak corresponding to the primary ($\alpha$) relaxation and a low-temperature peak corresponding to the secondary ($\beta$) relaxation. The intensity of the $\beta$ relaxation in $\rm {Pd}_{40}\rm {Cu}_{40}\rm {P}_{20}$ is almost 40$\%$ of that of the $\alpha$ relaxation, making it one of the strongest $\beta$ relaxations observed in metallic glasses. In contrast, $\rm {Pd}_{40}\rm {Ni}_{40}\rm {P}_{20}$ exhibits only an excess wing in the low-temperature range [20]. Panels f and h of Fig. 1 show the computed loss spectra of the two glasses from our DP DMS simulations. These spectra show remarkable consistency with the experimental results (Fig. 1e and g). To the best of our knowledge, this represents the first example in which experimental relaxation data of glasses are reproduced by atomic-level simulations.

**Figure 2.**
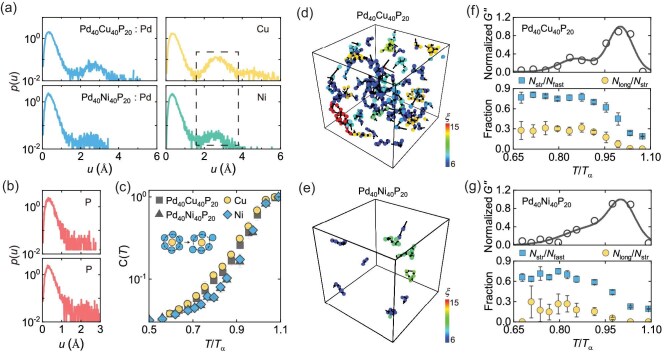
Structure rearrangements governing $\beta$ relaxation. (a and b) Probability distributions of atomic displacements $p(u)$ of Pd (blue), Cu/Ni (yellow/green) and P (red) atoms in $\rm {Pd}_{40}\rm {Cu}_{40}\rm {P}_{20}$ (upper panel) and $\rm {Pd}_{40}\rm {Ni}_{40}\rm {P}_{20}$ (lower panel), over an oscillation period $t_\omega = 100$ ns at 0.85$T_\alpha$ during DMS simulations. The dashed lines indicate the region corresponding to jumps between atoms. (c) Cage-breaking fraction $C(T)$ of all atoms (gray) as well as of Cu (yellow) and Ni (green) atoms over 100 ns. The inset illustrates the definition of cage breaking. (d and e) String-like motions at 0.85$T_\alpha$ for $\rm {Pd}_{40}\rm {Cu}_{40}\rm {P}_{20}$ and $\rm {Pd}_{40}\rm {Ni}_{40}\rm {P}_{20}$ glasses. The color scale represents the string size $\xi$, where strings with fewer than six atoms ($\xi < 6$) are not shown. (f and g) Correlation between relaxation and string-like motions in $\rm {Pd}_{40}\rm {Cu}_{40}\rm {P}_{20}$ and $\rm {Pd}_{40}\rm {Ni}_{40}\rm {P}_{20}$ glasses. In each panel, the upper plot shows the normalized loss modulus $G^{\prime \prime }$ as a function of reduced temperature ($T/T_\alpha$). The lower plot shows the fraction of string-like atoms among fast-moving atoms ($N_{\\smallriptsize\rm {str}} / N_{\\smallriptsize\rm {fast}}$, blue) and the fraction of atoms in long strings among all string-like atoms ($N_{\\smallriptsize\rm {long}} / N_{\\smallriptsize\rm {str}}$, yellow). Error bars represent the standard deviation across multiple simulation cycles.

Which structural rearrangements govern the $\beta$ relaxation? Our findings, detailed in Fig. 2 and Figs S9–S19 align with previous classical MD simulations. Specifically, atoms exhibit string-like motion as they escape their cages, a behavior observed in prior studies [50,66]. In the $\rm {Pd}_{40}\rm {Cu}_{40}\rm {P}_{20}$ glass, the high mobility of Cu atoms significantly increases the occurrence of string-like motion [67,68]. A recently proposed double-percolation scenario suggests that the percolation of mobile clusters, composed of these strings and other fast atoms, directly drives the $\beta$ relaxation (Figs S20–S24) [49,69,70]. A larger separation between the percolation thresholds of the slow and fast domains correlates with a lower activation energy for particle movement, as observed in $\rm {Pd}_{40}\rm {Cu}_{40}\rm {P}_{20}$ (Fig. S25). This observation is further supported by the differential activation energies derived from their diffusion coefficients and is consistent with the fragile-to-strong crossover commonly found in systems exhibiting well-defined $\beta$ relaxations (Fig. S12) [71,72]. Sun *et al.* [50] also reported that the $\rm {Al}_{90}\rm {Sm}_{10}$ metallic glass exhibits well-separated $\beta$ and $\alpha$ relaxations. Specifically, they identified a pronounced $\beta$-relaxation peak dominated by string-like motions, closely analogous to that observed in $\rm {Pd}_{40}\rm {Cu}_{40}\rm {P}_{20}$ glass. However, as shown in Fig. S23, mobile and immobile clusters exhibit slight differences in their fractal dimensions, with clusters in $\rm {Pd}_{40}\rm {Cu}_{40}\rm {P}_{20}$ showing a somewhat smaller fractal dimension; whether this corresponds to higher mobility remains to be investigated in future work.

We proceed to investigate the structural origin underlying the distinct relaxation behaviors of the two glasses. For this purpose, Voronoi analysis [73] is used to examine the short-range geometric features of both systems. The Voronoi index distributions are quite similar (Fig. S26), suggesting that the overall structural characteristics of the two glasses are nearly identical.

**Figure 3.**
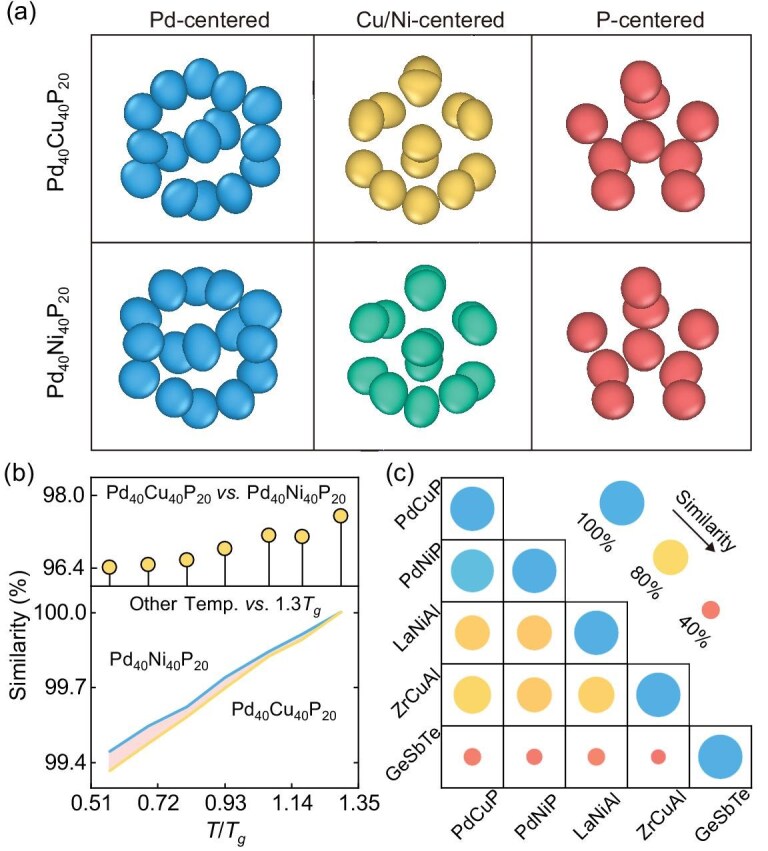
Geometric similarity analysis. (a) High-density regions of aligned Pd-centered, Cu/Ni-centered and P-centered clusters for $\rm {Pd}_{40}\rm {Cu}_{40}\rm {P}_{20}$ (upper) and $\rm {Pd}_{40}\rm {Ni}_{40}\rm {P}_{20}$ (lower) glasses. (b) Structural similarity derived from SOAP descriptors with $r_c=6.0$ Å. The upper panel shows the structural similarities between $\rm {Pd}_{40}\rm {Cu}_{40}\rm {P}_{20}$ and $\rm {Pd}_{40}\rm {Cu}_{40}\rm {P}_{20}$ glasses over a range of temperatures. The lower panel shows the similarities between $\rm {Pd}_{40}\rm {Cu}_{40}\rm {P}_{20}$ (yellow) and $\rm {Pd}_{40}\rm {Ni}_{40}\rm {P}_{20}$ (blue) during continuous cooling, relative to their respective structures at 1.3$T_g$. (c) Structural similarity matrix between different ternary model glasses at 300 K: PdCuP, $\rm {Pd}_{40}\rm {Cu}_{40}\rm {P}_{20}$; PdNiP, $\rm {Pd}_{40}\rm {Ni}_{40}\rm {P}_{20}$; LaNiAl, $\rm {La}_{50}\rm {Ni}_{35}\rm {Al}_{15}$; ZrCuAl, $\rm {Zr}_{46}\rm {Cu}_{46}\rm {Al}_{8}$; GeSbTe, $\rm {Ge}_{2}\rm {Sb}_{2}\rm {Te}_{5}$.

To further explore possible structural patterns, we employ the pairwise cluster-alignment strategy [74], a data-mining technique designed to identify and align atoms with similar packing motifs. This involves a preprocessing step in which the spatial distribution of each neighboring atom is calculated relative to the central atom. After alignment, consistent structures are identified using alignment scores below 0.20 (a smaller score indicates better alignment; compare Figs S31–S33). The high-density regions are then visualized as isosurfaces. Figure 3a displays such isosurfaces of the aligned clusters with a high degree of match, centered around the Pd, Cu/Ni and P atoms, respectively. Both glasses exhibit nearly identical aligned motifs, further reinforcing the strong similarity in their short-range orders.

We employ SOAP descriptors to encode the local atomic environments and quantify the structural similarity between the $\rm {Pd}_{40}\rm {Cu}_{40}\rm {P}_{20}$ and $\rm {Pd}_{40}\rm {Ni}_{40}\rm {P}_{20}$ glasses. The SOAP descriptors capture detailed atomic environments by expanding a Gaussian-smeared atomic density within a cutoff radius using spherical harmonics and radial basis functions [31], providing a robust framework for structural characterization of disordered systems. Based on these descriptors, the global similarity between two configurations is computed using the regularized-entropy match kernel, as implemented in the DScribe package [75,76]. Across the entire temperature range, a strong similarity between the $\rm {Pd}_{40}\rm {Cu}_{40}\rm {P}_{20}$ and $\rm {Pd}_{40}\rm {Ni}_{40}\rm {P}_{20}$ glasses is evident from the upper panel of Fig. 3b. While the similarity decreases somewhat with decreasing temperature, it remains consistently high, always exceeding 96.4%. At longer length scales, structural differences between the two glasses are further masked by intrinsic disorder, resulting in an even higher apparent similarity (Fig. S34).

The lower panel of Fig. 3b shows the structural evolution of each glass during the continuous cooling process, measured relative to their respective structures at 1.3$T_g$. Both glasses exhibit very high similarity (>99%), with $\rm {Pd}_{40}\rm {Ni}_{40}\rm {P}_{20}$ showing a smaller structural deviation than $\rm {Pd}_{40}\rm {Cu}_{40}\rm {P}_{20}$. This trend is consistent with their respective fragilities *m* (obtained from calorimetric measurements): 40 for $\rm {Pd}_{40}\rm {Ni}_{40}\rm {P}_{20}$ and 55 for $\rm {Pd}_{40}\rm {Cu}_{40}\rm {P}_{20}$, as shown in Table S3 and Fig. S7. For reference, Fig. 3c compares the structural similarity between the two Pd-based glasses with that of other typical glass systems ($\rm {La}_{50}\rm {Ni}_{35}\rm {Al}_{15}$ [25], $\rm {Zr}_{46}\rm {Cu}_{46}\rm {Al}_{8}$ [77] and $\rm {Ge}_{2}\rm {Sb}_{2}\rm {Te}_{5}$ [78]). These different metallic glasses have moderate structural similarity, while the similarities between the metallic glasses and the covalently bonded glass GeSbTe are notably lower. Further structural similarity analyses are provided in Fig. S34. In summary, the Voronoi analyses, cluster-alignment analyses and advanced featurization using SOAP all testify to a remarkable structural similarity between the two glasses. This shows that knowledge of atomic structure alone is insufficient to explain the distinct relaxation behaviors of the two glasses, emphasizing the need to include additional factors.

**Figure 4.**
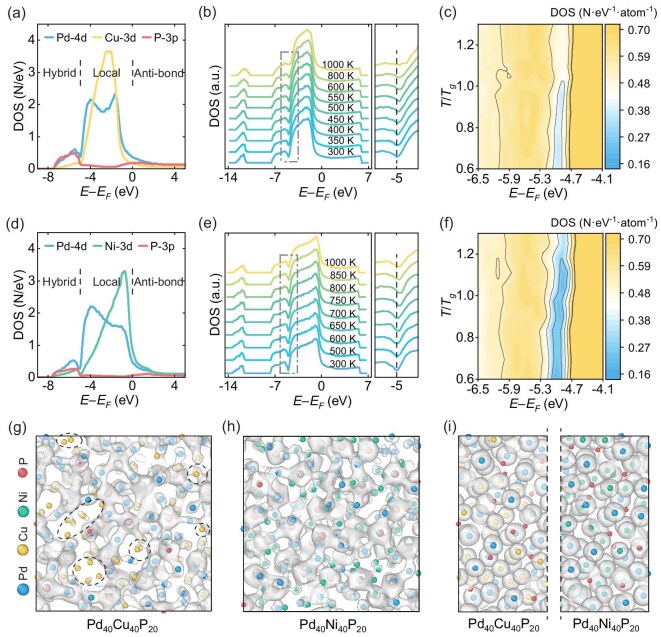
Density of states (DOS) and electronic distributions, demonstrating significant differences. Partial DOS for (a) $\rm {Pd}_{40}\rm {Cu}_{40}\rm {P}_{20}$ at 0.85$T_\alpha$. The electronic states are classified into three regions: ‘hybrid’, ‘local’ and ‘anti-bond’. (b) Total DOS during continuous cooling ($R = 2$ K/ns) of the $\rm {Pd}_{40}\rm {Cu}_{40}\rm {P}_{20}$ glass. The right panel highlights the progressive shift of the pseudo-gap towards lower-energy states. (c) Contour map of the total DOS around the pseudo-gap in $\rm {Pd}_{40}\rm {Cu}_{40}\rm {P}_{20}$. (d–f) The DOS and partial DOS results for $\rm {Pd}_{40}\rm {Ni}_{40}\rm {P}_{20}$ glass, using the same strategy as in (a–c). (g and h) Charge-density distribution (isosurface level = 0.15 *e*/Å^3^) within the ‘hybrid’ region, shown as 5-Å-thick slices for the $\rm {Pd}_{40}\rm {Cu}_{40}\rm {P}_{20}$ and $\rm {Pd}_{40}\rm {Ni}_{40}\rm {P}_{20}$ glasses. The dashed circles in (g) indicate Cu atoms in $\rm {Pd}_{40}\rm {Cu}_{40}\rm {P}_{20}$ glass. (i) Charge-density distribution (isosurface level = 0.30 *e*/Å^3^) within the ‘local’ region, shown as 5-Å-thick slices for the $\rm {Pd}_{40}\rm {Cu}_{40}\rm {P}_{20}$ (left) and $\rm {Pd}_{40}\rm {Ni}_{40}\rm {P}_{20}$ (right) glasses.

To uncover what determines the contrasting dynamic behaviors of the two glasses, we proceed to study their electronic structures and bonding characteristics (Figs S35–S40). Panels a and d of Fig. 4 show the partial electronic density of states (DOS). The region below roughly $-5$ eV (‘hybrid’) is dominated by strong orbital hybridization between the Pd-4d, Cu-3d and P-3p orbitals, reflecting covalent-like bonding interactions. From $-5$ eV to the Fermi energy $E_F$ (‘local’), the states primarily arise from the d orbitals of Pd and Cu, which are more localized to individual atoms and do not contribute to covalent-like bonding. Above $E_F$ (‘anti-bond’), the states correspond to anti-bonding orbitals.

Between the ‘hybrid’ and ‘local’ regions, a pseudo-gap emerges in both glasses. As the temperature decreases, as shown in panels b and e of Fig. 4, this pseudo-gap gradually deepens and shifts towards lower energy. As shown in panels c and f of Fig. 4, the pseudo-gap forms earlier for $\rm {Pd}_{40}\rm {Ni}_{40}\rm {P}_{20}$, in fact already at 1.3$T_g$. Similar behavior is observed across different quenching rates (Figs S41 and S42). The early formation of a pseudo-gap indicates that the electronic structure in $\rm {Pd}_{40}\rm {Ni}_{40}\rm {P}_{20}$ quickly becomes energetically stabilized during cooling. This shows that the bonding interactions in $\rm {Pd}_{40}\rm {Ni}_{40}\rm {P}_{20}$ are more robust and well defined.

To compare the bonding interactions in $\rm {Pd}_{40}\rm {Ni}_{40}\rm {P}_{20}$ and $\rm {Pd}_{40}\rm {Cu}_{40}\rm {P}_{20}$, we plot, in panels g and h of Fig. 4, the charge density distributions in the ‘hybrid’ region (below $-5$ eV). Orbital hybridization in this energy range gives rise to numerous Pd–P and Cu/Ni–P covalent-like bonds, collectively forming a global covalent network. Comparing the two systems, $\rm {Pd}_{40}\rm {Ni}_{40}\rm {P}_{20}$ exhibits a more uniform and interconnected covalent network than $\rm {Pd}_{40}\rm {Cu}_{40}\rm {P}_{20}$, which has a sparser structure with more isolated Cu atoms (Fig. 4g). In contrast, in the ‘local’ region, the electrons are highly localized around the Pd and Cu/Ni atoms.

**Figure 5.**
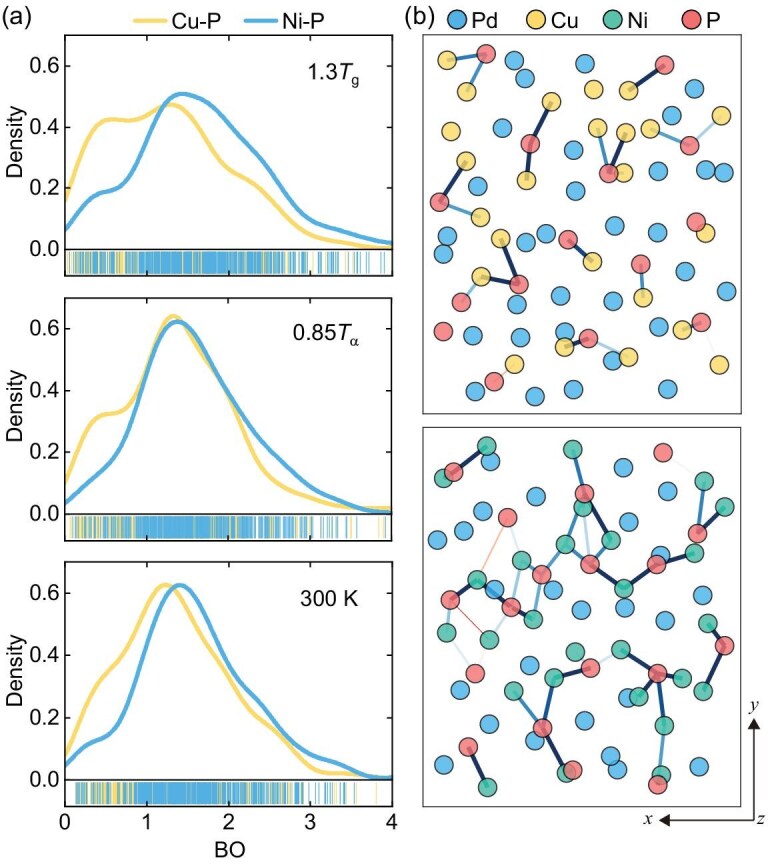
Bond order. (a) Density distributions of BOs for neighboring Cu–P and Ni–P bonds at 1.3$T_g$, 0.85$T_\alpha$ and 300 K. The bar codes at the bottom represent data density. (b) 5-Å-thick slices of the atomic configurations, together with the Cu–P and Ni–P BOs, projected onto the *x*-*y* plane for $\rm {Pd}_{40}\rm {Cu}_{40}\rm {P}_{20}$ (upper panel) and $\rm {Pd}_{40}\rm {Ni}_{40}\rm {P}_{20}$ (lower panel) at 0.85$T_\alpha$. Filled circles represent atoms, and colored lines indicate BOs. Thicker and darker lines indicate stronger BOs for the Cu–P or Ni–P bonds (the upper limit for the BO color scale is set to 1.5).

Next the bond orders (BOs) between nearest-neighbor atoms identified by Voronoi tessellation analysis are considered (Figs S43–S46). The density-derived electrostatic and chemical (DDEC6) method is employed, providing an advanced computational framework for determining BOs in a wide range of materials [73,79,80]. Bond order quantifies the strength of chemical bonds between atoms, with larger BO values indicating stronger bonds. Figure 5a compares the BO distributions for Cu–P and Ni–P bonds at three typical temperatures (1.3$T_g$, 0.85$T_\alpha$ and 300 K). A distinct pre-peak on the low-BO side is observed for Cu–P bonds, with an intensity significantly higher than that of Ni–P bonds in the same region, demonstrating the presence of numerous weak Cu–P bonds. Moreover, the primary peak position of the BO distribution for Cu–P bonds is slightly lower than that for Ni–P bonds, indicating that Cu–P bonds are generally weaker than Ni–P bonds.

Figure 5b provides a structural visualization of BOs between P atoms and their nearest-neighbor Cu/Ni atoms for both model glasses. Thicker and darker lines indicate larger BOs for the Cu/Ni–P bonds. Clearly, $\rm {Pd}_{40}\rm {Cu}_{40}\rm {P}_{20}$ has fewer strong Cu–P bonds than the Ni–P bonds in $\rm {Pd}_{40}\rm {Ni}_{40}\rm {P}_{20}$, which is consistent with the results discussed above. We focus on the Cu/Ni–P bonds because the Pd–P bonds primarily serve to form and stabilize the overall bonding network and remain largely immobile, whereas other types of bonds are fewer in number and generally weaker (as detailed in Fig. S45). Figure S46 further presents the distributions of average BOs and of Cu–P BOs around Cu/Ni atoms with the 10% largest and 10% smallest displacements, again highlighting the correlation between bond strength and atomic mobility.

The formation and early stabilization of a relatively uniform and strong bonding network in $\rm {Pd}_{40}\rm {Ni}_{40}\rm {P}_{20}$ results in a reduced atomic mobility that limits the ability of atoms to undergo large-scale rearrangements. Consequently, this system has fewer mobile regions available for independent $\beta$ relaxation. Such a strong bonding network and reduced atomic mobility can notably enhance the GFA. Robust hybridization and the bonding network stabilize the liquid-like structure and promote resistance to crystallization, enabling $\rm {Pd}_{40}\rm {Ni}_{40}\rm {P}_{20}$ to form glasses more readily. In contrast, $\rm {Pd}_{40}\rm {Cu}_{40}\rm {P}_{20}$, with its delayed pseudo-gap formation and weaker bonding network, exhibits increased atomic mobility that facilitates crystallization. This explains the pronounced $\beta$ relaxation and the relatively poor GFA of $\rm {Pd}_{40}\rm {Cu}_{40}\rm {P}_{20}$ compared with $\rm {Pd}_{40}\rm {Ni}_{40}\rm {P}_{20}$. Consistent with experiments [65], strong orbital hybridization and early stabilization of bonding interactions contribute to a more rigid local atomic environment in $\rm {Pd}_{40}\rm {Ni}_{40}\rm {P}_{20}$. However, it remains unclear whether, and how, the long-range heterogeneity of weak bonds contributes to the relaxation behavior and GFA, which warrants further investigation.

## CONCLUSIONS

To summarize, by means of deep-learning-based interatomic potentials, we have explored the intricate relationship between atomic structure, chemical bonding and relaxation dynamics in two Pd-based metal-metalloid glasses. By combining structural and electronic analyses, we have uncovered that key differences in bonding interactions may strongly affect the dynamic properties. Our findings demonstrate the necessity of incorporating electronic structure analysis for a proper understanding of metallic-glass dynamics, and they show why even small variations in structure can lead to significant differences in glass properties.

## METHODS

### Experiments

Master alloy ingots were prepared by induction melting high-purity elements ($\ge 99.95\%$) under a Ti-gettered argon atmosphere and re-melted six times for homogeneity. Glassy ribbons were produced by melt-spinning the alloys onto a cold copper roller at 40–60 m/s under argon. Transmission electron microscopy samples were prepared by ion milling and thinned for high-angle annular dark-field imaging using a JEOL JEM-2100F microscope.

Dynamic mechanical spectroscopy was performed using a TA Q-800 analyzer in film tension mode under nitrogen at 1 Hz with a heating rate of 3 K/min. Differential scanning calorimetry was carried out on a Mettler Toledo DSC3 at 20 K/min under nitrogen, while flash DSC measurements were conducted on a Mettler Toledo Flash DSC 2+ equipped with a MultiSTAR UFS1 sensor under an argon flow of 80 mL/min.

### *Ab initio* molecular dynamics

All *ab initio* molecular dynamics (AIMD) simulations were performed using VASP [81] within the NVT ensemble with a Nosé–Hoover thermostat. Ion–electron interactions were described by the PAW method [82], and exchange–correlation effects were treated using the PBE functional [83]. A plane-wave cutoff energy of 450 eV and an electronic convergence criterion of $10^{-4}$ eV were adopted. The *k*-point mesh was chosen according to the supercell size (Tables S1 and S2). A time step of 5 fs was used.

### Deep potentials

Deep potential (DP) models for the PdNi(Cu)P systems were constructed using the DeePMD-kit package [56,84,85]. The DeepPot-SE descriptor was adopted to ensure translational, rotational and permutational invariance, with a cutoff radius of 6.0 Å. The embedding network consisted of layers with 25, 50 and 100 neurons, while the fitting network contained three hidden layers with 240 neurons each. The learning rate decayed from 0.001 to $3.51 \times 10^{-8}$ every 5000 steps. Initial loss prefactors for energy, force and virial were set to 0.02, 1000 and 200, respectively, and gradually adjusted to 1 during training.

### DP-driven molecular dynamics simulations

Systems containing 500 and 8000 atoms were melted at 2000 K and annealed at 1000 K for 100 ns until energy stabilization, followed by cooling to 200 K at rates of 2, 10 and 100 K/ns. Simulations were performed using LAMMPS [86,87] with DP integration under periodic boundary conditions and a Nosé–Hoover thermostat in the NPT ensemble with a time step of 2 fs.

For DMS simulations, a sinusoidal strain $\varepsilon (t) = \varepsilon _0 \sin (2\pi t / t_\omega )$ was applied along the *x* direction in the NVT ensemble, with oscillation periods $t_\omega$ of 1, 10 and 100 ns and a time step of 10 fs. A strain amplitude of 1.0% ensured the linear-response regime, and the resulting stress was fitted to obtain the storage and loss moduli.

### Electronic interaction calculations

Electronic structure calculations were performed on 500-atom glassy supercells obtained from DP-driven cooling simulations. The density of states, charge density distributions and bond orders were calculated using a $2 \times 2 \times 2$  *k*-point mesh, with other parameters consistent with the AIMD settings. Bond orders were analyzed using the Chargemol framework [80].

## Supplementary Material

nwag006_Supplemental_File
