# Supplementary material for: Beyond geometry orders: uncovering bonding-heterogeneity-dominated structure-relaxation coupling in glasses
Source: Natl Sci Rev. 2026 Jan 15;13(5):nwag006. doi: 10.1093/nsr/nwag006 (PMC12908929; doi:10.1093/nsr/nwag006)
Supplement: nwag006_Supplemental_File [file nwag006_supplemental_file.pdf]

Beyond geometry orders: Uncovering bonding heterogeneity dominated structure-relaxation coupling in glasses
Supplemental Material

Liang Gao[1], Jia-Qi Gao[1], Qing-Zhou Bu[1], Qun Yang[1,3], Yang Sun[4], Kai-Ming Ho[5], Qi Wang[2,*] Jeppe C. Dyre[6,†] and Hai-Bin Yu[1‡]

[1] *Wuhan National High Magnetic Field Center and School of Physic, Huazhong University of Science and Technology, Wuhan 430074, China*
[2] *Institute of Materials, China Academy of Engineering Physics, Mianyang 621908, China*
[3] *College of Physics and Electronic Engineering, Chongqing Normal University, Chongqing 401331, China*
[4] *Department of Physics, Xiamen University, Xiamen 361005, China*
[5] *Department of Physics and Astronomy, Iowa State University, Ames, Iowa 50011, United States and*
[6] *Glass and Time, IMFUFA, Department of Science and Environment, Roskilde University, DK-4000 Roskilde, Denmark*

This document provides details on the PdCu(Ni)P Deep Potentials, Experiments, DP-driven Molecular Dynamics Simulations, Structural Features, and First Principles Calculations. Figure overview:

- **Sec.1 Construction of Deep Potentials**
  1. Workflow of DP - Fig. S1
  2. Data sets - Table. S1, Table. S2, and Fig. S2
  3. Tests of PdCu(Ni)P DPs - Figs. S3 and Fig. S4
- **Sec.2 Experiments**
  1. XRD - Fig. S5
  2. DMA - Fig. S6
  3. DSC - Fig. S7 and Table. S3
- **Sec.3 DP-driven Molecular Dynamics Simulations**
  1. Preparation of glasses and $g(r)$ - Fig. S8
  2. ISF and MSD - Fig. S9, Fig. S10, Fig. S11, Fig. S13, and Fig. S14
  3. DMS and motion mode - Fig. S15, Fig. S16, Fig. S17, Fig. S18, and Fig. S19
  4. Double-percolation scenario - Fig. S20, Fig. S21, Fig. S22, Fig. S23, Fig. S24, and Fig. S25
- **Sec.4 Structural Features**
  1. Voronoi analysis - Fig. S26 and Fig. S27
  2. Bond-angle and bond-length distributions - Fig. S28 and Fig. S29
  3. Soild-like network - Fig. S30
  4. Alignment and similarity - Fig. S31, Fig. S32, Fig. S33, and Fig. S34
- **Sec.5 Electronic Insights**
  1. DOS and pseudo-energy gap - Fig. S35, Fig. S36, Fig. S37, Fig. S38, Fig. S39, Fig. S40, and Fig. S41
  2. Bader charge analysis and electronic assignment - Fig. S43 and Fig. S44
  3. Bond orders - Fig. S45 and Fig. S46

All microstructure images were generated using OVITO[1].

---

* qwang_mse@caep.cn
† dyre@ruc.dk
‡ haibinyu@hust.edu.cn

CONSTRUCTION OF DEEP POTENTIALS

Figure S1 illustrates the workflow for constructing DPs and conducting DP-driven molecular dynamics simulations, exemplified by the Pd-Cu-P system (a similar procedure applies to Pd-Ni-P). The workflow is divided into three stages: Dataset collection, DP Training, and DP-driven molecular dynamics simulations.

i) Dataset collection. In order to obtain a realistic interaction potential, 17 compositions within the Pd-Cu-P ternary phase diagram were studied by *ab initio* molecular dynamics (AIMD) simulations to generate the dataset (Fig. S1(a) and (b), Table. S1). Crystalline single-element and binary compounds were simulated at 300 K for 12 ps. Liquid Pd and Cu are prepared and sampled at high temperatures (2000 K and 1600 K, respectively), with a duration of 24 ps. Amorphous $Pd_{40}Cu_{40}P_{20}$ structures at different temperatures were obtained by continuously cooling a 2000 K liquid $Pd_{40}Cu_{40}P_{20}$ at rate 10000 K/ns. At each intermediate temperature, from 2000 K to 200 K in 200 K intervals, the sample was annealed for an additional 70 ps for adequate structural sampling. Additionally, amorphous $Pd_{40}Cu_{40}P_{20}$ structures at 200 K, 400 K, and 600 K, scaled within a range of -10% to 10%, were also prepared. In all cases, NVT simulations were performed for these structures at the specified temperatures to obtain a large dataset of configurations, as well as their energies and forces [2]. The final datasets for Pd-Cu-P and Pd-Ni-P contain 306,000 and 158,000 configurations, respectively.

ii) DP Training. As illustrated in Fig. S1(c), in the framework of DPs [3–6], the local environment matrices R_i are constructed from the input structure and go through an embedding network and a fitting network to yield the prediction of E_i. The forces are then obtained as the negative gradient of the energy with respect to atomic positions. Here, R_i contains the coordinates of all neighboring j atoms within a cut-off radius r_c relative to atom i. A weighting function s_{ij} relative to the radial distance r_{ij} is used to reduce the components of R_i to $\hat{R}_i$ (e.g., $x_{ij} \rightarrow \hat{x}_{ij} = s_{ij}x_{ij}/r_{ij}$), and to ensure a smooth decay from 0 to r_c. Thereafter, s_{ij} is transformed into a local embedding matrix g_i through the embedding network. The g_i and $\hat{R}_i$ form the local feature matrix D_i, which preserves the translation, permutation and rotation symmetries. Subsequently, D_i is processed through a multi-layer fitting network to predict the energy E_i^{ω}. The total energies E, forces F_i, and virial stresses Ξ_i are obtained from the predictions and compared with the DFT-derived values. The neural network weights and other tunable parameters are updated based on the loss function. Once all iterations are complete and the performance on the validation sets converges, the final PdNi(Cu)P DPs are obtained. Our PdNi(Cu)P DPs achieve high prediction accuracy on the validation sets, with an energy RMSE of 3.98 meV/atom and a force RMSE of 0.082 eV/Å for PdCuP, and an energy RMSE of 7.43 meV/atom and a force RMSE of 0.089 eV/Å for PdNiP. Detailed comparison plots are presented in Figs. S3 and Fig. S4.

iii) DP-driven molecular dynamics simulations. The PdNi(Cu)P DPs are then applied to run molecular dynamics simulations (Fig. S1(d)), including the liquid-melt cooling to prepare glass samples and DMS simulations to investigate relaxation behaviors. Large systems comprising 8000 atoms are employed, with simulations extending over long time scales (hundreds of nanoseconds). Figure S1(e) presents the potential energy evolution of $Pd_{40}Cu_{40}P_{20}$ glass during cooling at rates of 100, 10 and 2 K/ns (represented by blue, yellow and red curves, respectively), with the corresponding glass transition temperatures (T_g) determined to be 620 K, 600 K and 580 K, respectively.

Figure S1(f) shows a diagram illustrating the DMS simulation strategy. In DMS simulations, a sinusoidal strain $\varepsilon(t) = \varepsilon_0 \sin(\omega t)$ is applied along the x-direction of the simulation box. The stress, i.e., the response of the simulated system to the applied strain, is fitted to $\sigma(t) = \sigma_0 \sin(\omega t + \delta)$. Thereafter, the loss spectrum of system is calculated from $G'' = \sigma_0/\varepsilon_0 \sin(\delta)$.

DP-DRIVEN MOLECULAR DYNAMICS SIMULATIONS

Recently, a double-percolation scenario was proposed that links the α and β relaxation processes in disordered materials to the percolation of immobile and mobile particles, respectively[7]. Following this framework, Figure S20(a) and (b) present a double-percolation analysis of $Pd_{40}Cu_{40}P_{20}$ and $Pd_{40}Ni_{40}P_{20}$ glasses, providing further insight into their relaxation dynamics. The results show that the distinct separation of the percolations of mobile and immobile particles leads to a pronounced β relaxation in $Pd_{40}Cu_{40}P_{20}$. In contrast, the closer proximity of the two percolations results in a stronger coupling of α and β relaxations in $Pd_{40}Ni_{40}P_{20}$, suppressing a well-defined β relaxation. As an example, we present two percolated clusters that span the configuration space at the characteristic temperatures of α and β relaxation for $Pd_{40}Cu_{40}P_{20}$ glass in Fig. S20(c) and (d).

STRUCTURAL FEATURES

Previous reports have mentioned that the MRO linked by P-centered polyhedra in $Pd_{40}Ni_{40}P_{20}$ glass is the major contributor to its strong GFA. Building on this, Fig. S30 (a) shows slices of the structural network in $Pd_{40}Cu_{40}P_{20}$

glass composed of P-centered <0 3 6 0>, <0 2 8 0>, <0 4 4 0> polyhedra and their neighboring atoms at $1.3T_g$ and $0.85T_\alpha$. This network represents the solid-like structure in $Pd_{40}Cu_{40}P_{20}$ glass and is the source of its viscosity. It can be observed that this network is denser at $0.85T_g$ compared to $1.3T_\alpha$, with a noticeable reduction in voids. This indicates an increase in MRO and viscosity following the glass transition. Fig. S30 (d) exhibits the string-like motion on the solid-like network in $Pd_{40}Cu_{40}P_{20}$ glass. Fig. S30 (c) shows the high-angle annular dark-field (HADDF) micro-graphs of as-cast $Pd_{40}Cu_{40}P_{20}$ and $Pd_{40}Ni_{40}P_{20}$ at room temperature, where the dark regions represent liquid-like areas within the amorphous structure, while the bright regions are solid-like areas. This is consistent with the simulated results in Fig. S30 (a) and (b).

Fig. S30 (b) apply the same strategy as Fig. S30 (a) for $Pd_{40}Ni_{40}P_{20}$ glass. There are a few notable differences: i) In Fig. S30 (b), the network slices at the two temperatures show much smaller voids, and the density of the network is visually higher. ii) The distinction between the network slices at the two temperatures is minimal, with only a slight narrowing of the void regions. Certainly, the simulated results remain consistent with the HADDF micro-graph. Considering the definition of fragility m, the differences in the solid-like networks of both glasses before and after the glass transition are reasonable. The degree of network change in $Pd_{40}Cu_{40}P_{20}$ glass is greater than that in $Pd_{40}Ni_{40}P_{20}$ glass, which can be indirectly interpreted as indicating that the viscosity gradient in $Pd_{40}Cu_{40}P_{20}$ glass is higher than that in $Pd_{40}Ni_{40}P_{20}$ glass at the glass transition. This is consistent with the results shown in Figure 4(d) lower panel in the main text. Undoubtedly, the glass-forming ability arises from their solid-like networks, which facilitate the formation of stable glassy states from glass forming liquids during rapid quenching.

ELECTRONIC INSIGHTS

In the Bader charge partitioning, Fig. S43, Ni atoms and P atoms in $Pd_{40}Ni_{40}P_{20}$ glass deviate more from the uncharged state than in $Pd_{40}Cu_{40}P_{20}$ glass. This implies that Ni atoms have more electronic interactions with P atoms than Cu and P. Based on the Bader boundaries, shown in Fig. S43 (b), the electrons in the orbital hybridization region are assigned. Fig. S43 (c) illustrates the result of the normalized to the total number of electrons in the orbital hybridization region. The results indicate that Ni atoms in $Pd_{40}Ni_{40}P_{20}$ glass contribute more electrons to the covalent network in conjunction with P atoms compared to the contribution from Cu atoms and P atoms in $Pd_{40}Cu_{40}P_{20}$ model.

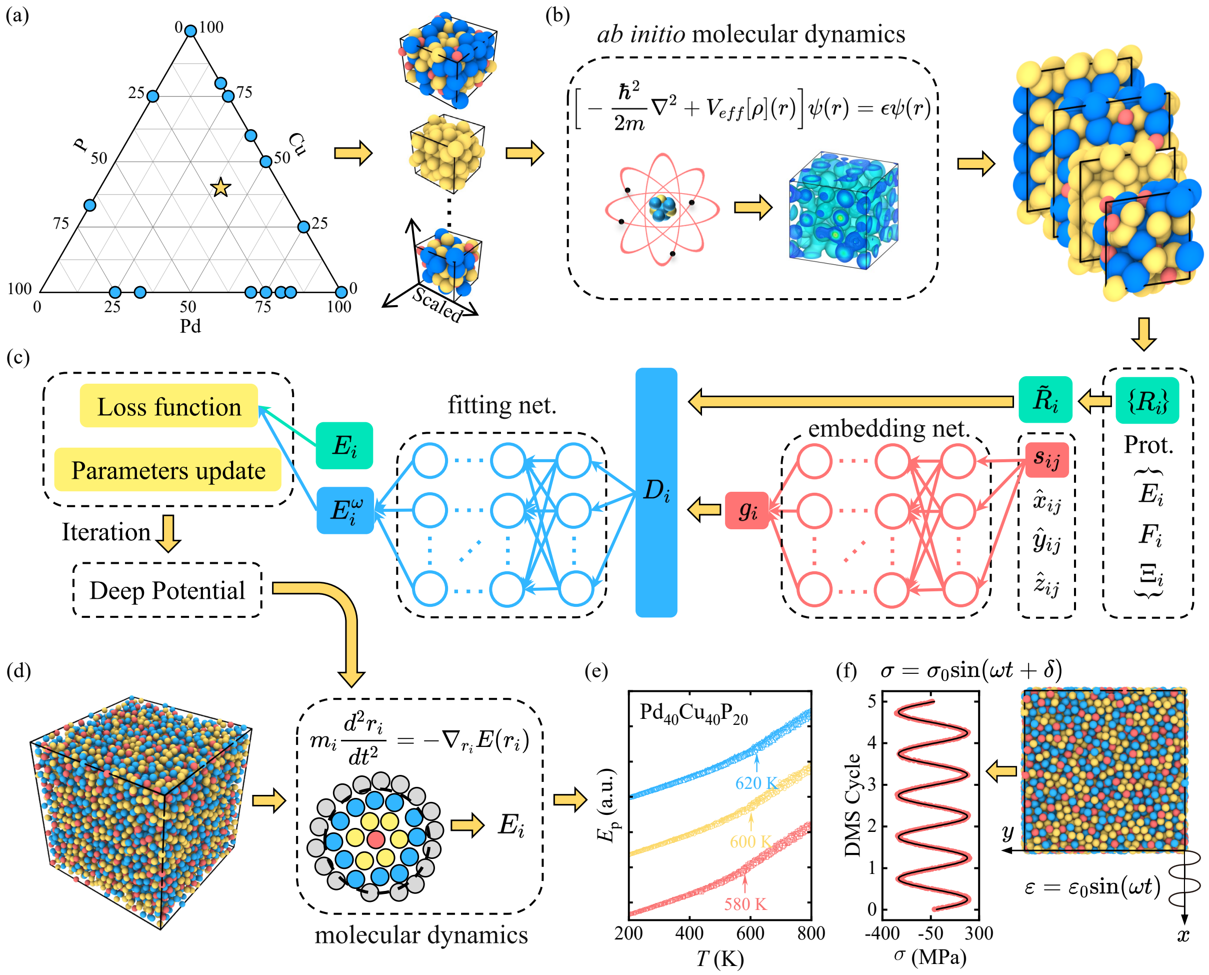

FIG. S1. A workflow of DP construction and DP-driven molecular dynamics, exemplified by the $Pd_{40}Cu_{40}P_{20}$ glass. Dataset collection: (a) A diverse set of initial structures, containing both crystalline and amorphous phases. (b) A series of configuration snapshots are obtained via *ab initio* molecular dynamics using the initial structures. Training: (c) The process starts by constructing local environment matrices R_i, where atoms within a cutoff radius r_c around atom i are selected as inputs, and atomic properties (energy E_i, force F_i, virial Ξ_i) as targets. The generalized coordinates $\widetilde{R}_i$ and the local embedding matrix g_i (derived from the weighting function s_{ij}), together form the local feature matrix $D_i = (g_{i1})^T \hat{R}_i(\hat{R}_i)^T g_{i2}$, which is then passed to the fitting network to predict the energy E_i^ω. Finally, the loss function is computed, and neural network parameters are iteratively optimized until the DPs are finalized. DP-driven simulation: (d) The trained DPs are used to describe the potential energy surface of large systems containing thousands of atoms. The atomic energy E_i is determined based on the local environment of atom i. (e) The evolution of potential energy during continuous cooling at rates R of 100, 10 and 2 K/ns (blue, yellow and red), and the T_gs are identified as 620 K, 600 K and 580 K, respectively. (f) The DMS simulation strategy. A sinusoidal strain $\varepsilon = \varepsilon_0 \sin(\omega t)$ is applied along the x-direction of simulation box, and the resulting stress response is fitted by $\sigma = \sigma_0 \sin(\omega t + \delta)$. The phase difference δ between σ and ε represents the internal friction of the system.

TABLE S1. The training and validation set of PdCuP DP

Systems	The number of atoms in the box/k-mesh	Temperatures (K)	Time (ps)	The number of snapshots in training set	The number of snapshots in validation set	Notes
$Pd_{40}Cu_{40}P_{20}$	$64/3 \times 3 \times 3$	2000-0	200	40000	0	
		2000	70	12000	2000	
		1800	70	12000	2000	
		1600	70	12000	2000	
		1400	70	12000	2000	
		1200	70	12000	2000	
		1000	70	12000	2000	
		800	70	12000	2000	
		600	70	12000	2000	
			160	32000	0	Scaled
		400	70	12000	2000	
			160	32000	0	Scaled
		200	70	12000	2000	
			160	32000	0	Scaled
Pd_2Cu_2P	$160/2 \times 2 \times 2$	300	12	2000	400	
		300-1800	50	10000	0	
Pd	$108/3 \times 3 \times 3$	300	12	2000	400	
		2000	24	4000	800	
Cu	$108/3 \times 3 \times 3$	300	12	2000	400	
		1600	24	4000	800	
$CuPd_3$	$128/2 \times 2 \times 4$	300	12	2000	400	
CuPd	$128/3 \times 3 \times 3$	300	12	2000	400	
Cu_3Pd_2	$160/3 \times 3 \times 2$	300	12	2000	400	
Cu_3Pd	$108/3 \times 3 \times 3$	300	12	2000	400	
Cu_4Pd	$160/3 \times 3 \times 2$	300	12	2000	400	
Cu_3P	$96/2 \times 2 \times 4$	300	12	2000	400	
CuP_2	$96/3 \times 3 \times 2$	300	12	2000	400	
PdP_3	$128/2 \times 2 \times 4$	300	12	2000	400	
PdP_2	$96/3 \times 3 \times 3$	300	12	2000	400	
Pd_7P_3	$120/2 \times 2 \times 2$	300	12	2000	400	
Pd_3P	$128/3 \times 3 \times 2$	300	12	2000	400	
Pd_4P	$120/3 \times 2 \times 3$	300	12	2000	400	
$Pd_{15}P_2$	$102/2 \times 4 \times 2$	300	12	2000	400	
Total			1670	306000	28000	

TABLE S2. The training and validation set of PdNiP DP

Systems	The number of atoms in the box/k-mesh	Temperatures (K)	Time (ps)	The number of snapshots in training set	The number of snapshots in validation set	Notes
$Pd_{40}Ni_{40}P_{20}$	$64/3 \times 3 \times 3$	2000-0	100	20000	0	
		2000	35	6000	1000	
		1800	35	6000	1000	
		1600	35	6000	1000	
		1400	35	6000	1000	
		1200	35	6000	1000	
		1000	35	6000	1000	
		800	35	6000	1000	
		600	35	6000	1000	
			80	16000	0	Scaled
		400	35	6000	1000	
			80	16000	0	Scaled
		200	35	6000	1000	
			80	16000	0	Scaled
Pd	$108/3 \times 3 \times 3$	300	12	2000	400	
		2000	24	4000	800	
Ni	$108/3 \times 3 \times 3$	300	6	1000	200	
		2000	12	2000	400	
PdP_3	$128/3 \times 2 \times 2$	300	12	2000	400	
PdP_2	$96/3 \times 2 \times 2$	300	12	2000	400	
Pd_7P_3	$120/2 \times 2 \times 2$	300	12	2000	400	
Pd_3P	$128/3 \times 3 \times 2$	300	12	2000	400	
Pd_4P	$120/3 \times 2 \times 3$	300	12	2000	400	
$Pd_{15}P_2$	$102/2 \times 4 \times 2$	300	12	2000	400	
NiP	$96/3 \times 3 \times 3$	300	6	1000	200	
NiP_2	$96/2 \times 4 \times 2$	300	6	1000	200	
Ni_3P	$128/3 \times 3 \times 3$	300	6	1000	200	
Ni_2P	$108/3 \times 3 \times 3$	300	6	1000	200	
$Ni_{12}P_5$	$136/2 \times 4 \times 3$	300	6	1000	200	
Ni_5P_4	$144/3 \times 3 \times 3$	300	6	1000	200	
Pd_3Ni	$96/3 \times 2 \times 2$	300	6	1000	200	
PdNi	$96/3 \times 2 \times 2$	300	6	1000	200	
Total			859	158000	16000	

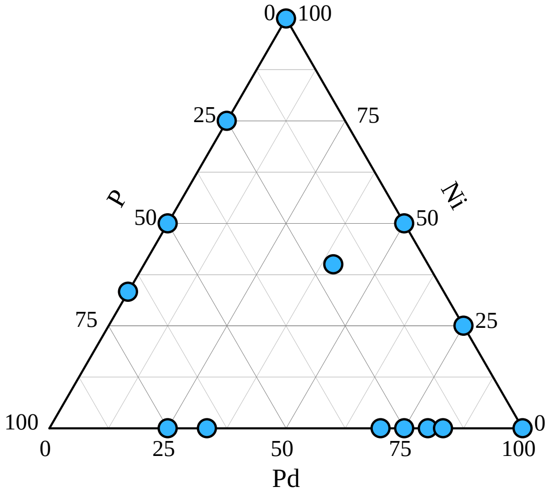

FIG. S2. The composition of the dataset for PdNiP Deep Potential, containing both crystalline and amorphous phases (some amorphous phases are scaled).

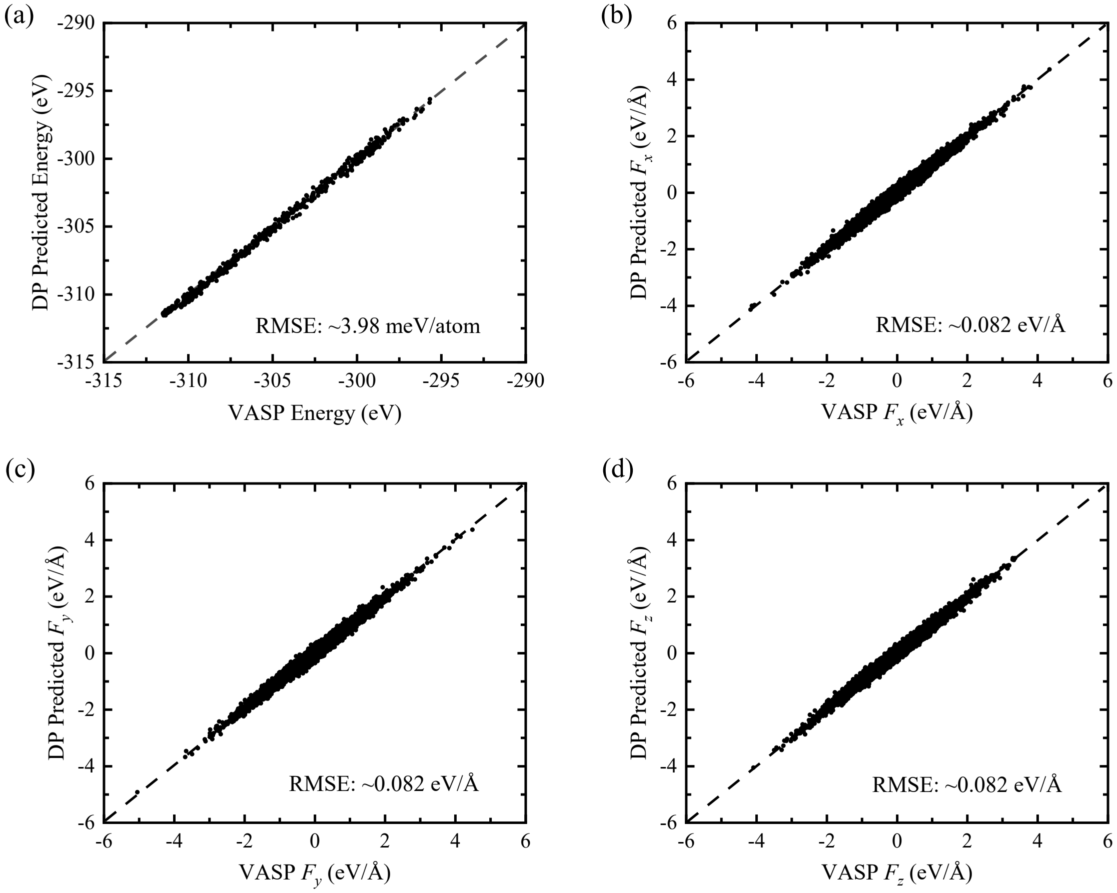

FIG. S3. Tests of PdCuP Deep potential. Comparison of energy and force (three directions) predicted by PdCuP DP and calculated from first principles using VASP.

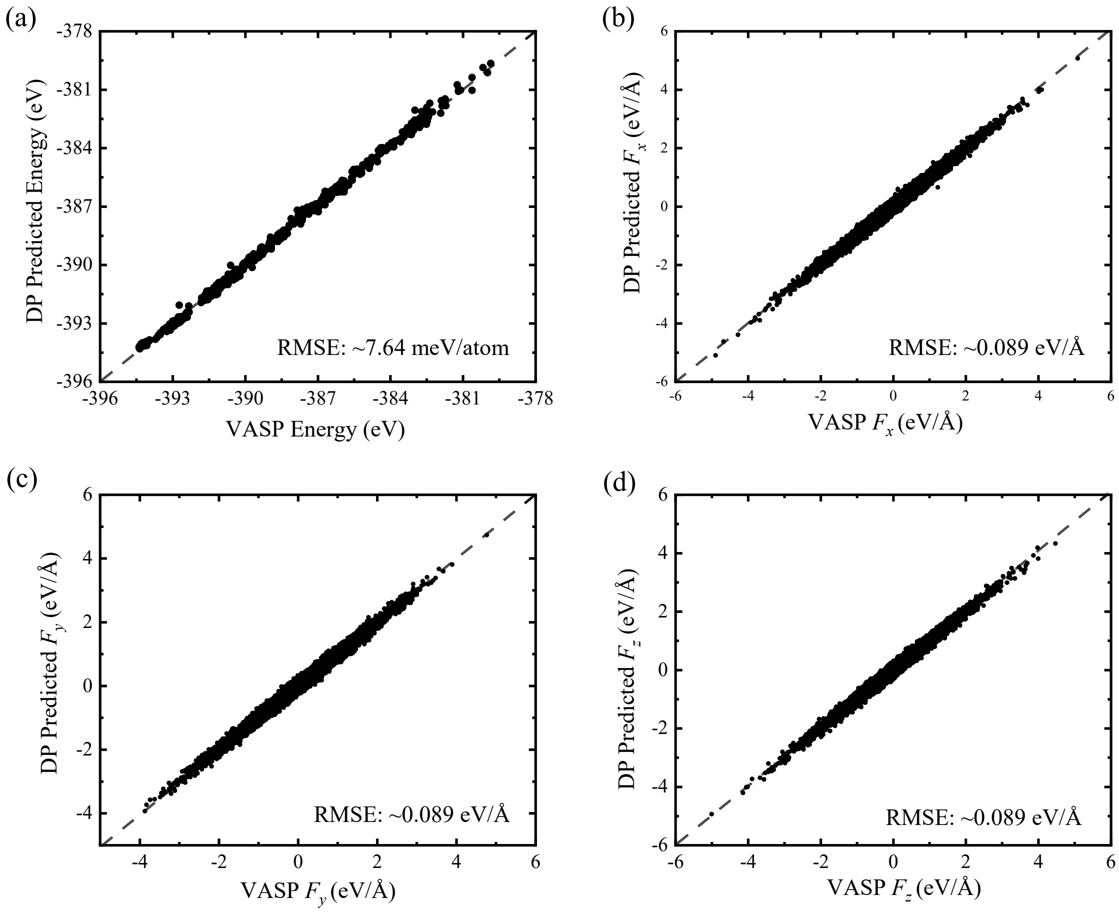

FIG. S4. Tests of PdNiP Deep potential. Comparison of energy and force (three directions) predicted by PdNiP DP and calculated from first principles using VASP.

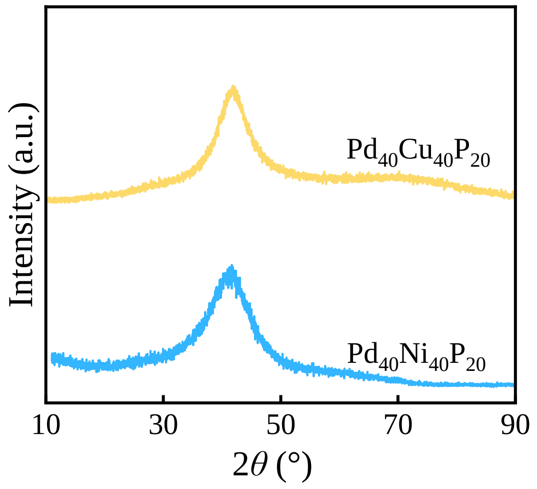

FIG. S5. X-ray diffraction patterns of as-cast $Pd_{40}Cu_{40}P_{20}$ (yellow) and $Pd_{40}Ni_{40}P_{20}$ (blue) glass ribbons.

TABLE S3. Details of DSC (40 K/min)

	$Pd_{40}Cu_{40}P_{20}$	$Pd_{40}Ni_{40}P_{20}$
T_g (K)	530	580
T_x (K)	576	655
T_m (K)	860	872
T_l (K)	901	898
m	55	40

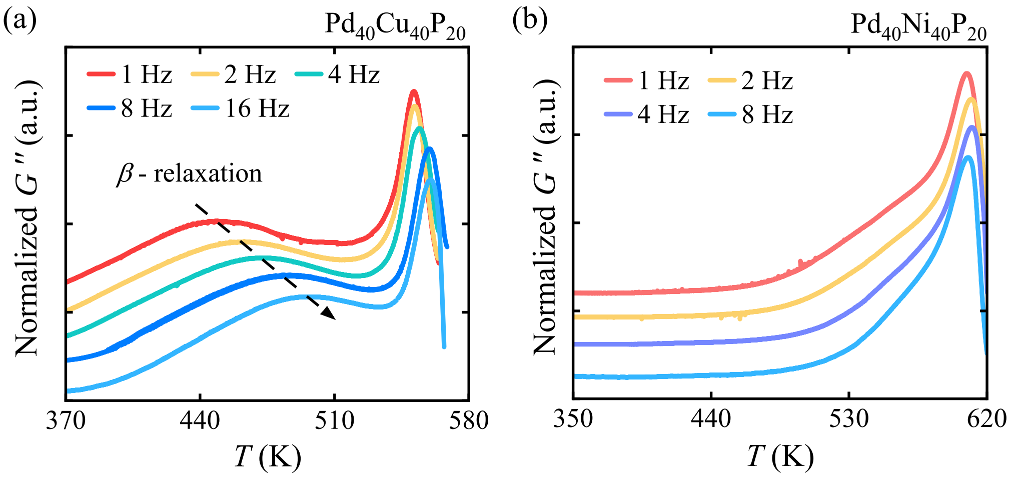

FIG. S6. Dynamic Mechanical Analysis (DMA) of as-cast (a) $Pd_{40}Cu_{40}P_{20}$ and (b) $Pd_{40}Ni_{40}P_{20}$ glass ribbons over a range of frequencies.

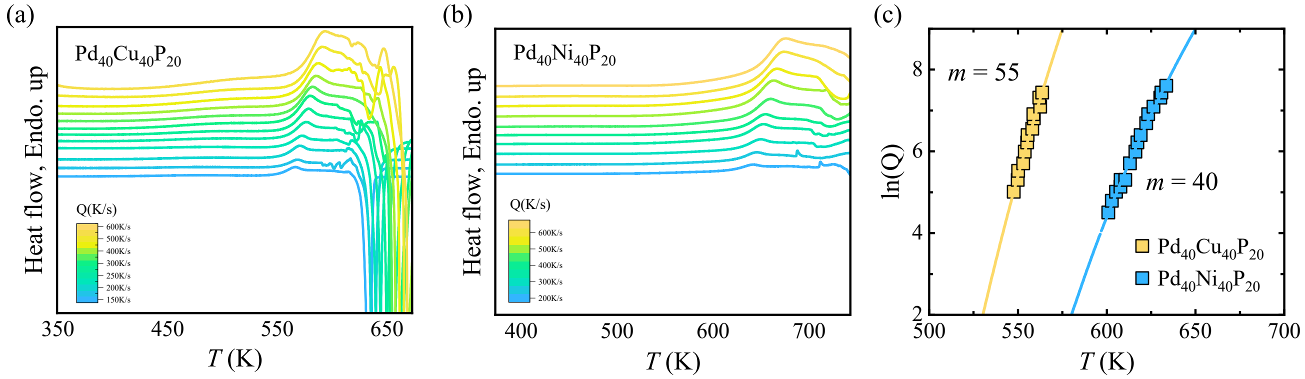

FIG. S7. Heat flow curves of as-cast (a) $Pd_{40}Cu_{40}P_{20}$ and (b) $Pd_{40}Ni_{40}P_{20}$ glass ribbons span a wide range of heating rates, using Flash Differential Scanning Calorimetry (FDSC). (c) VFT fitting for heating rate Q and glass transition temperature dependence T_g. The estimated fragility m is 55 and 40, respectively.

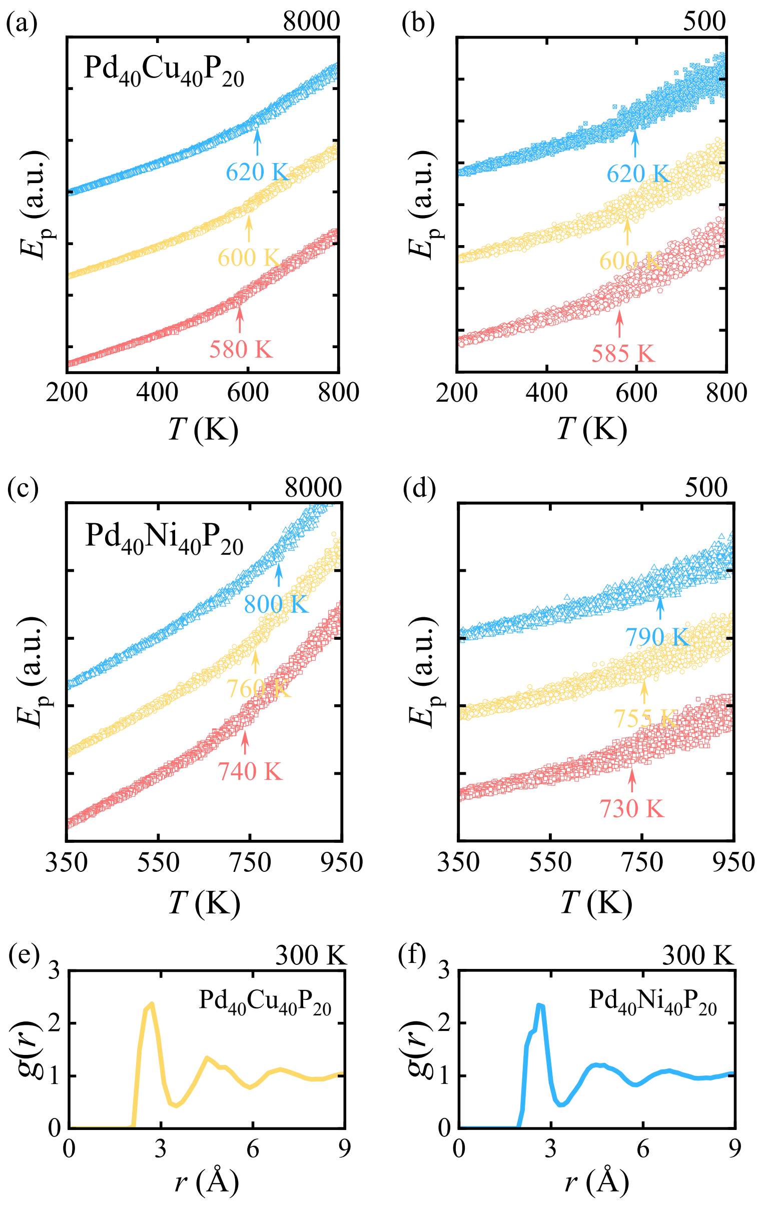

FIG. S8. Potential energy evolution of 8000-atom and 500-atom (a-b) $Pd_{40}Cu_{40}P_{20}$ and (c-d) $Pd_{40}Ni_{40}P_{20}$ glasses during continuous cooling with different cooling rates R (blue: 100 K/ns; yellow: 10 K/ns; red: 2 K/ns). Radial distribution function of (e) $Pd_{40}Cu_{40}P_{20}$ and (f) $Pd_{40}Ni_{40}P_{20}$ glasses at 300 K.

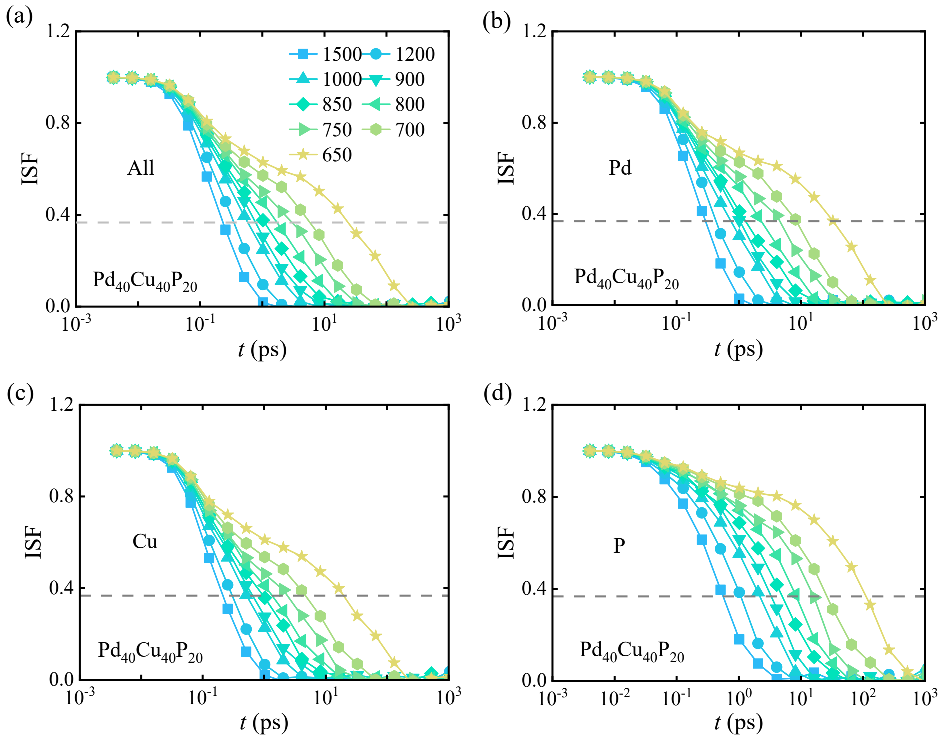

FIG. S9. The Intermediate Scatter Functions (ISF) of $Pd_{40}Cu_{40}P_{20}$ glass over a range of temperatures.

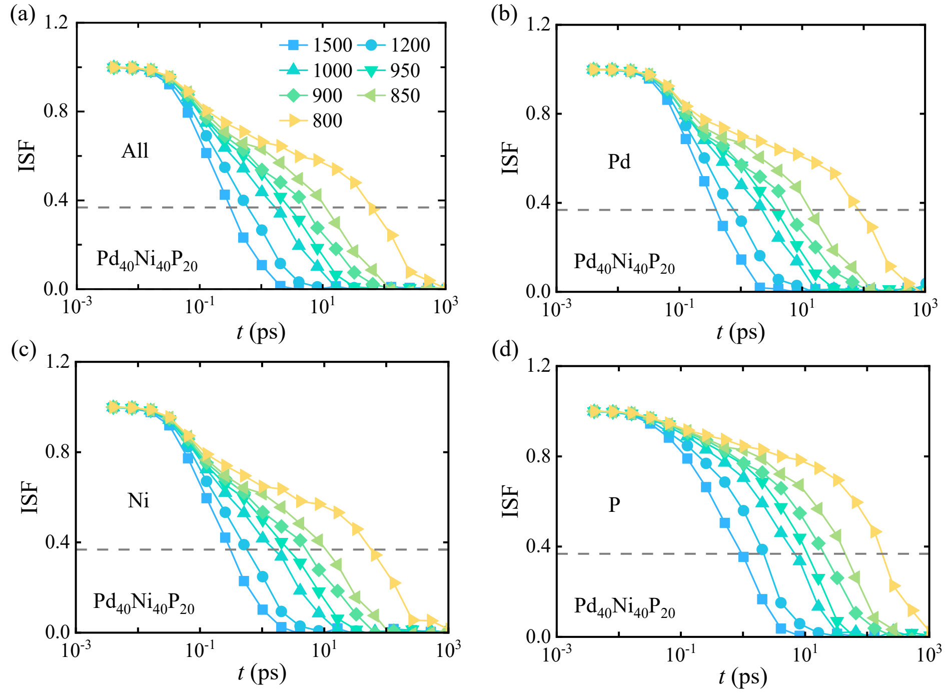

FIG. S10. The Intermediate Scatter Functions (ISF) of $Pd_{40}Ni_{40}P_{20}$ glass over a range of temperatures.

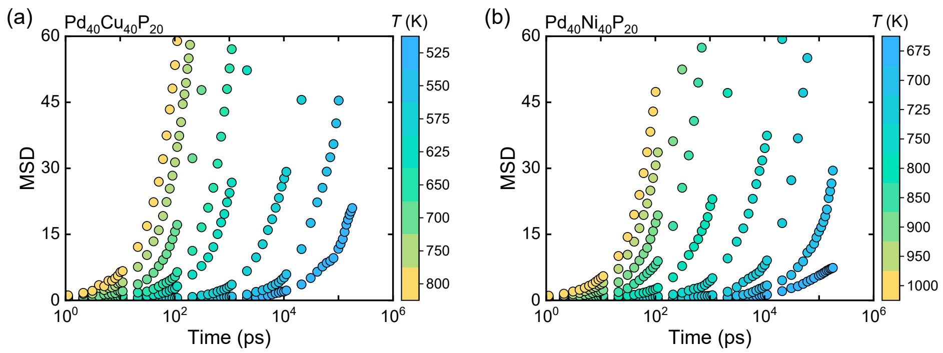

FIG. S11. The Mean Square Displacement (MSD) of (a) $Pd_{40}Cu_{40}P_{20}$ and (b) $Pd_{40}Ni_{40}P_{20}$ glasses over a range of temperatures.

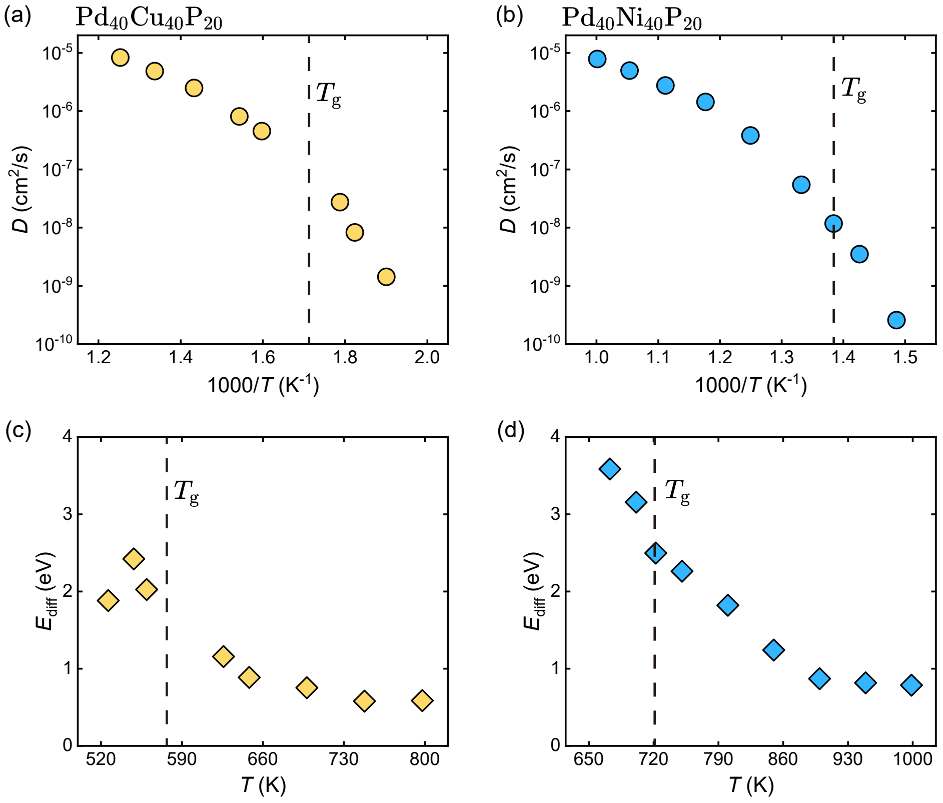

FIG. S12. (a-b) Average diffusion coefficient and (c-d) differential activation energy of $Pd_{40}Cu_{40}P_{20}$ (right) and $Pd_{40}Ni_{40}P_{20}$ (left) glasses.

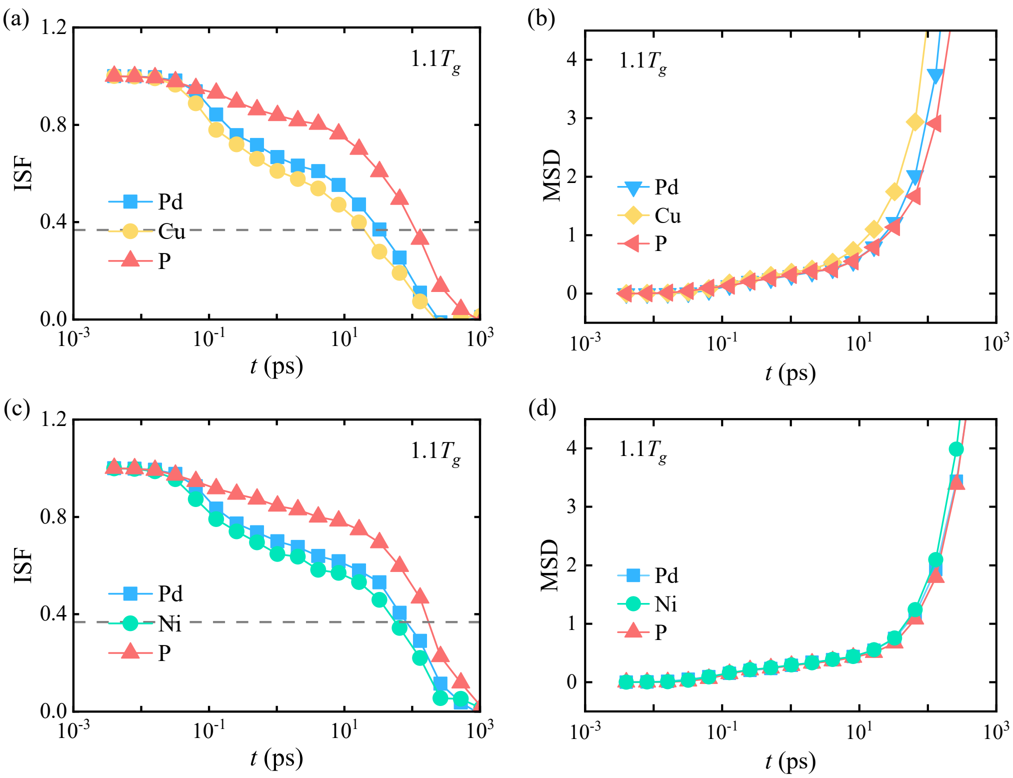

FIG. S13. The Intermediate Scatter Functions (ISF) and Mean Square Displacement (MSD) of $Pd_{40}Cu_{40}P_{20}/Pd_{40}Ni_{40}P_{20}$ glass at $1.1T_g$.

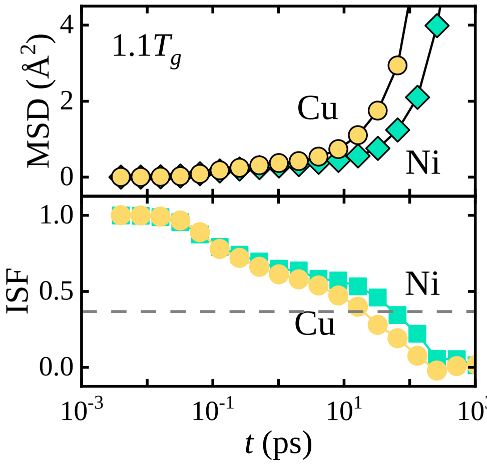

FIG. S14. The Intermediate Scatter Functions (ISF) and Mean Square Displacement (MSD) of Cu/Ni atoms in $Pd_{40}Cu_{40}P_{20}/Pd_{40}Ni_{40}P_{20}$ glass at $1.1T_g$.

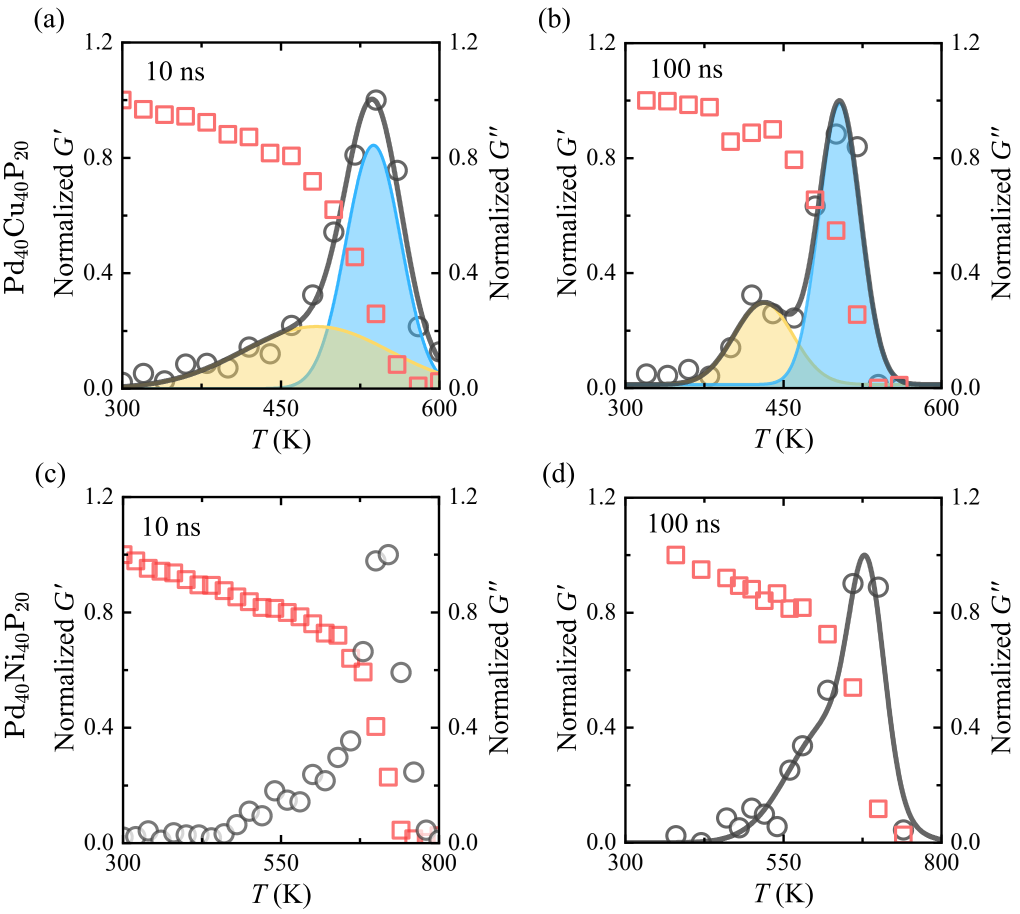

FIG. S15. Dynamic Mechanical Spectroscopy (DMS) simulation of (a-b) $Pd_{40}Cu_{40}P_{20}$ and (c-d) $Pd_{40}Ni_{40}P_{20}$ glasses with oscillation period $t_\omega = 10$, 100 ns. The loss moduli G'' of $Pd_{40}Cu_{40}P_{20}$ glass is fitted by a series of Gaussian peaks.

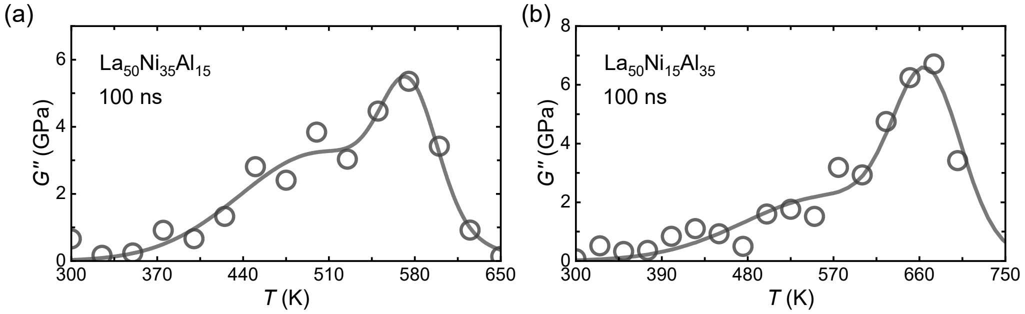

FIG. S16. Dynamic Mechanical Spectroscopy (DMS) simulation of (a) $La_{50}Ni_{35}Al_{15}$ and (b) $La_{50}Ni_{15}Al_{35}$ glasses with oscillation period $t_\omega = 100$ ns. The β-relaxation strength of $La_{50}Ni_{15}Al_{35}$ is weaker than that of $La_{50}Ni_{35}Al_{15}$, opposite to the trend of Al–Al covalent-like bonds [8]. This suggests that in La–Ni–Al metallic glasses, bonds and their interactions play a similar role as those discussed in the present manuscript, i.e., enhancing glass-forming ability while suppressing relaxation.

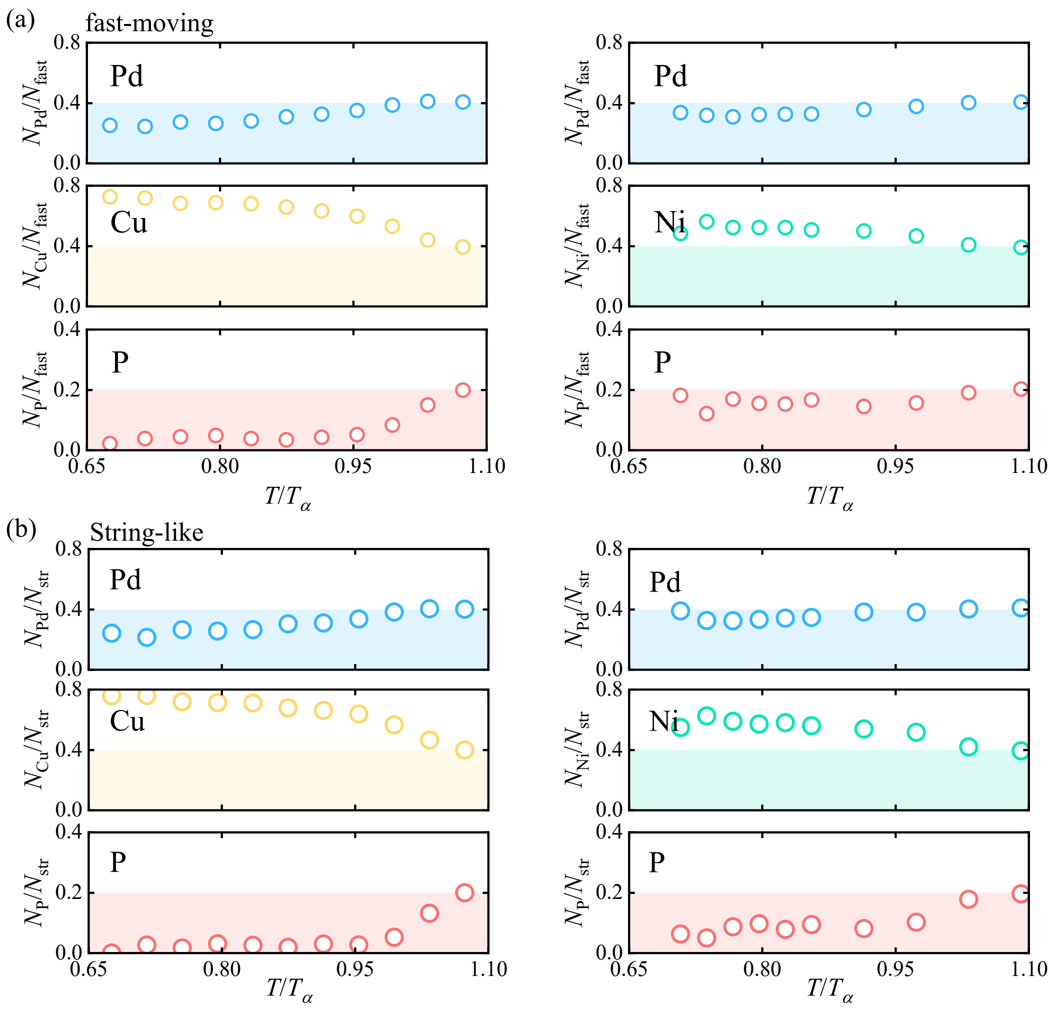

FIG. S17. The percentage of chemical composition in (a) fast-moving atoms and (b) string-like atoms of $Pd_{40}Cu_{40}P_{20}$ (left) and $Pd_{40}Ni_{40}P_{20}$ (right) glasses. The upper boundary of the coloured region is the percentage of that component in the system (e.g., Pd: 0.4 in $Pd_{40}Cu_{40}P_{20}$).

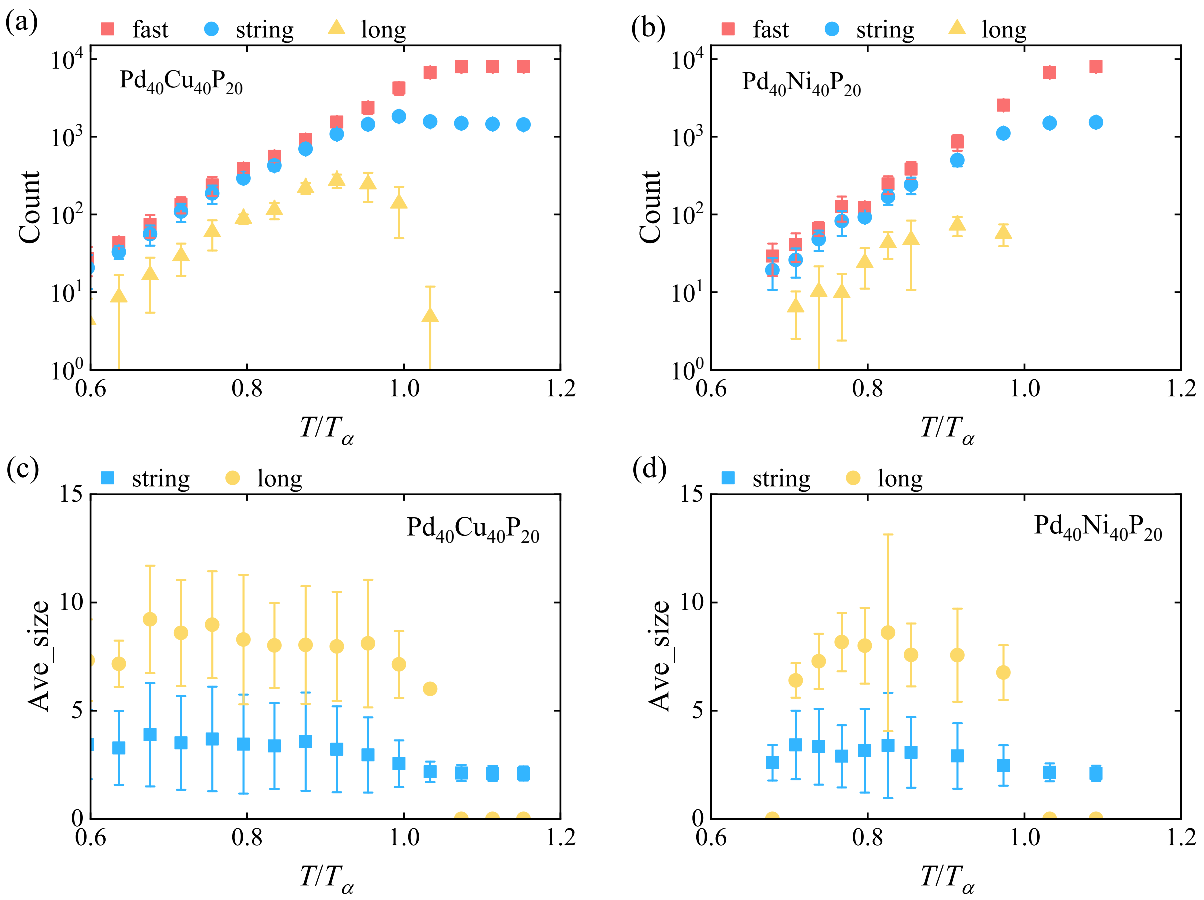

FIG. S18. The number of atoms with different motion modes (fast: red; string: blue; long-string: yellow) during continuous cooling of (a) $Pd_{40}Cu_{40}P_{20}$ and (b) $Pd_{40}Ni_{40}P_{20}$ glasses. (c-d) The average size of string-like motions and long-string motions for both glasses.

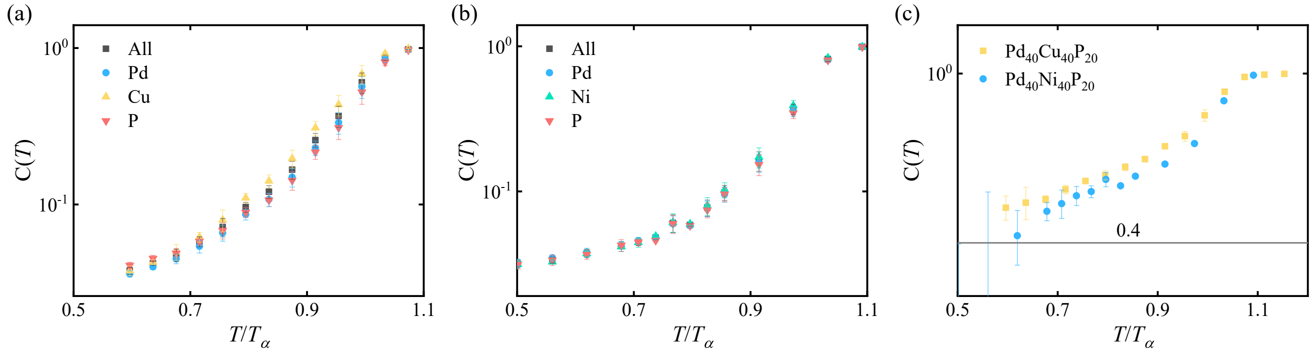

FIG. S19. The cage-breaking fraction $C(T, t)$ of (a) $Pd_{40}Cu_{40}P_{20}$ and (b) $Pd_{40}Ni_{40}P_{20}$ glasses. (c) Cage-breaking fraction of string-like motion atoms in both glasses. Notably, string-like motion resulted in the loss of at least 40% of the neighbors.

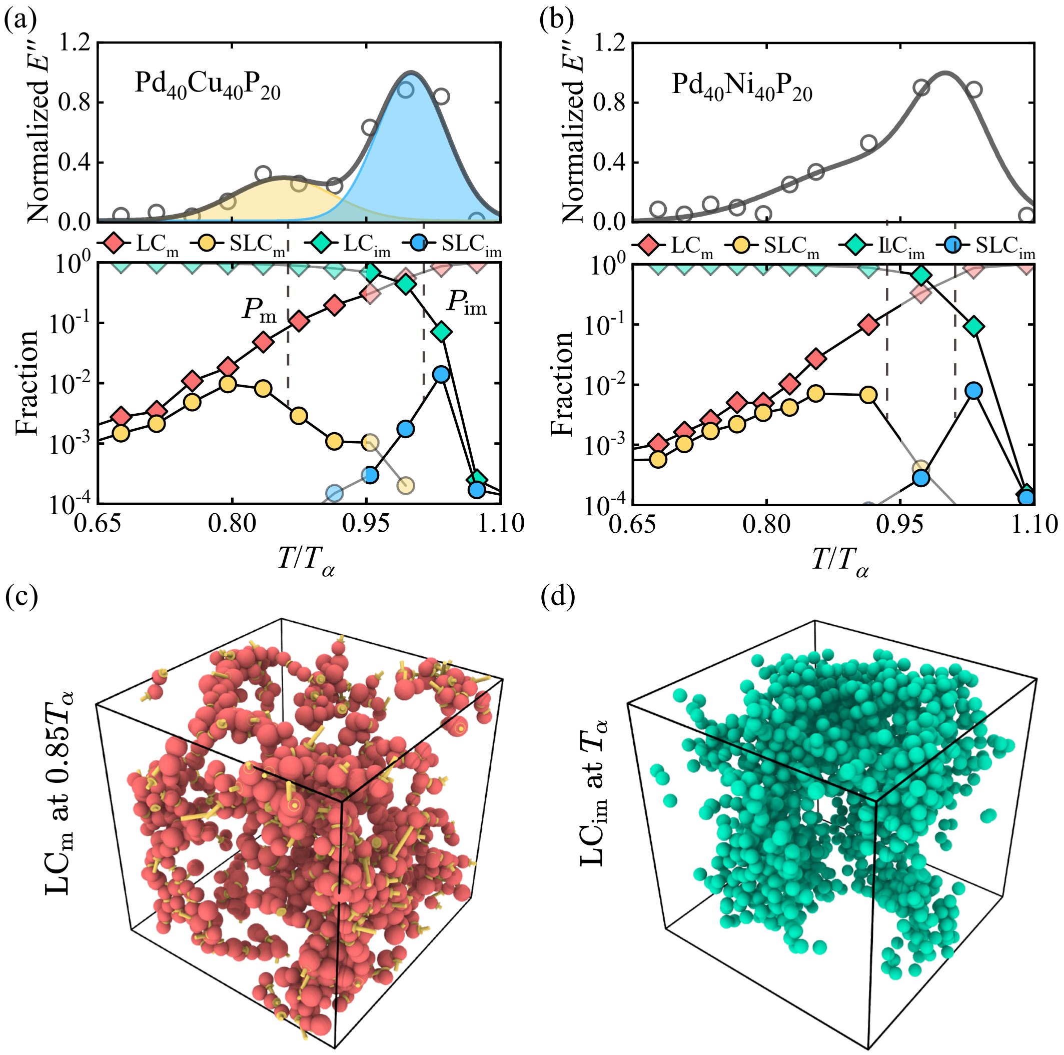

FIG. S20. Double-percolation scenario in (a) $Pd_{40}Cu_{40}P_{20}$ and (b) $Pd_{40}Ni_{40}P_{20}$ glasses.

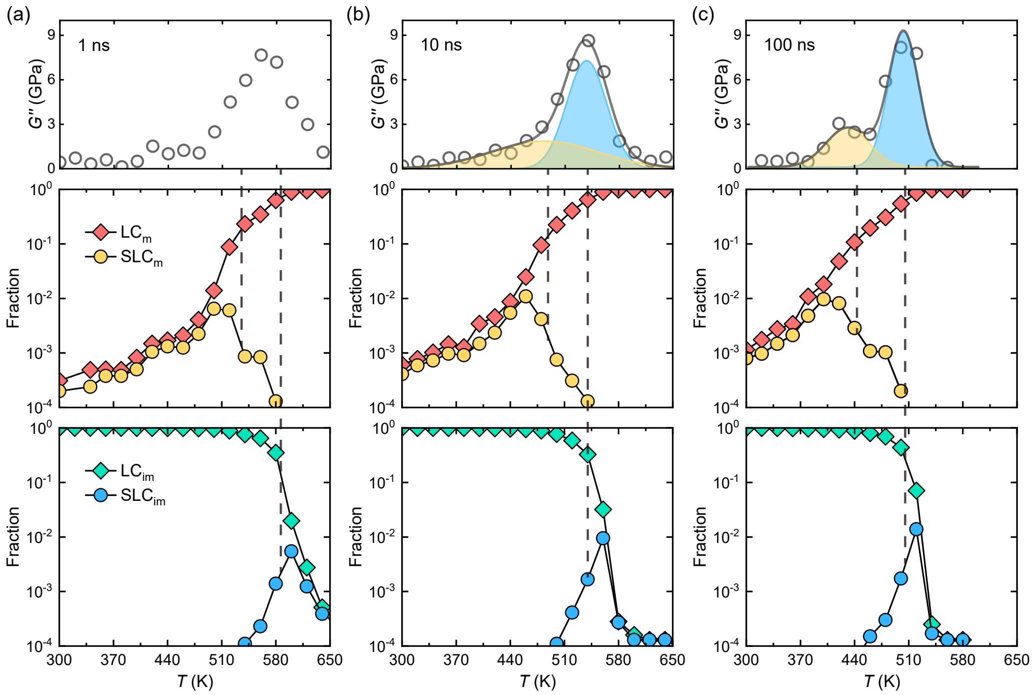

FIG. S21. Dynamic Mechanical Spectroscopy simulation and Double-Percolation analysis of $Pd_{40}Cu_{40}P_{20}$ glass with oscillation periods t_ω = 1, 10, 100 ns.

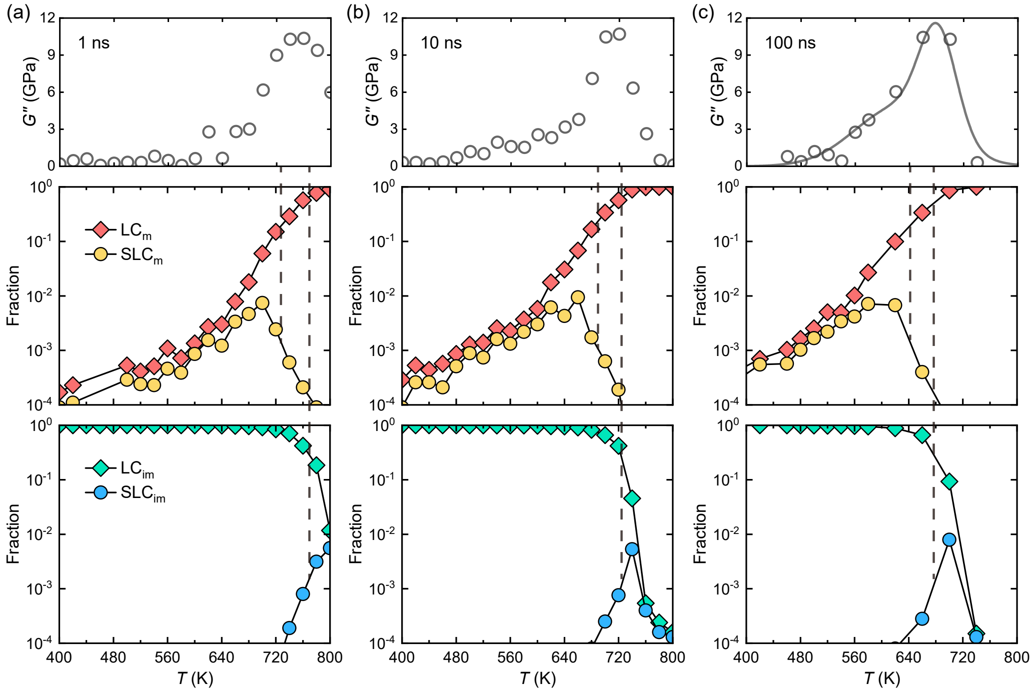

FIG. S22. Dynamic Mechanical Spectroscopy simulation and Double-Percolation analysis of $Pd_{40}Ni_{40}P_{20}$ glass with oscillation periods t_ω = 1, 10, 100 ns..

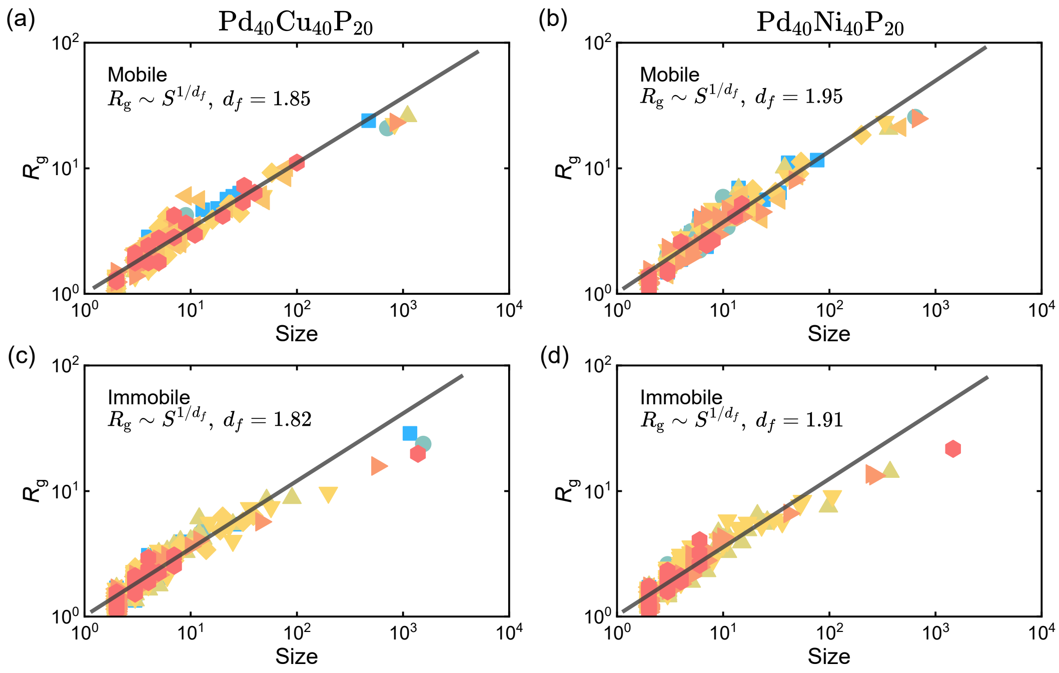

FIG. S23. The radius of gyration Rg with size of (a-b) mobile and (c-d) immobile clusters for $Pd_{40}Cu_{40}P_{20}$ (right) and $Pd_{40}Ni_{40}P_{20}$ (left) glasses with different oscillation periods and temperature. In $Pd_{40}Cu_{40}P_{20}$, both mobile and immobile clusters exhibit slightly smaller fractal dimensions compared with those in $Pd_{40}Ni_{40}P_{20}$. This suggests that the spatial geometry of dynamic clusters differs between the two glasses. Whether the smaller fractal dimension contributes to stronger dynamical heterogeneity or to the characteristics of the β relaxation remains an open question that requires further investigation.

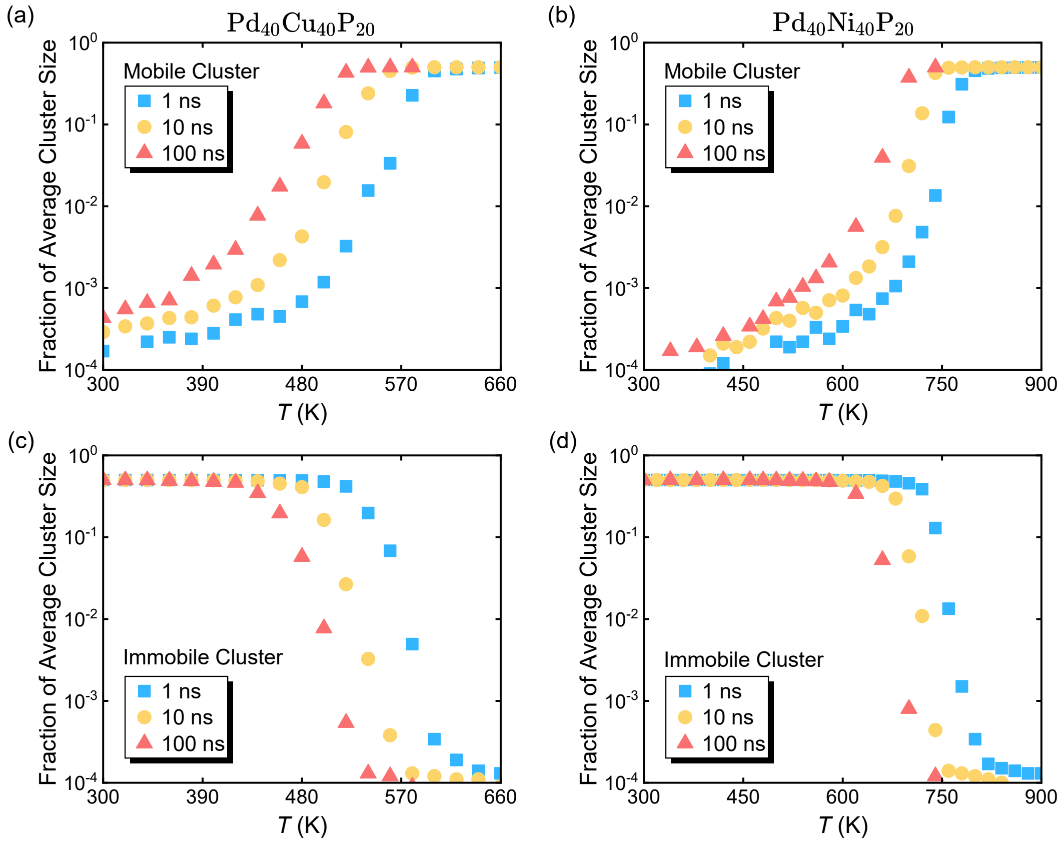

FIG. S24. The fraction of average cluster size of (a-b) mobile and (c-d) immobile of $Pd_{40}Cu_{40}P_{20}$ (right) and $Pd_{40}Ni_{40}P_{20}$ (left) glasses over a range of oscillation periods t_ω.

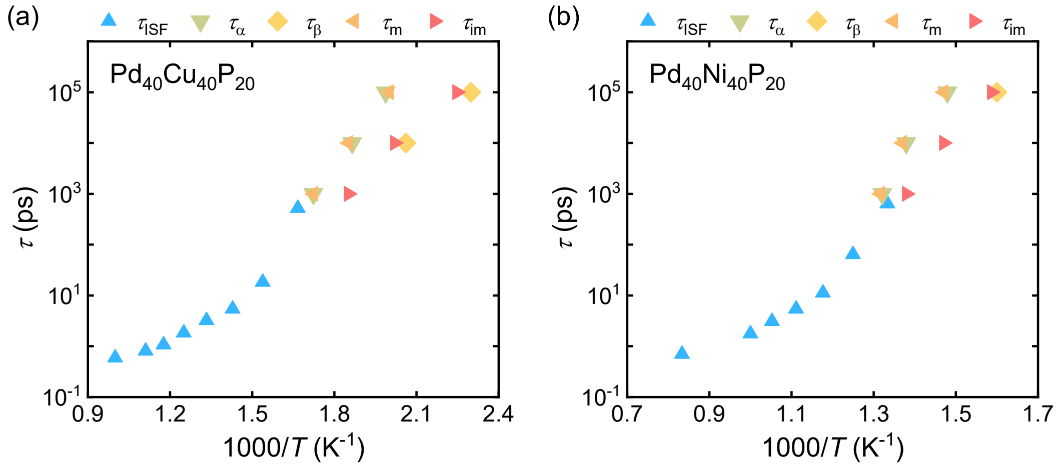

FIG. S25. The temperature-dependent relaxation time map of (a) $Pd_{40}Cu_{40}P_{20}$ and (b) $Pd_{40}Ni_{40}P_{20}$ glasses. $\tau_{\rm ISF}$: relaxation time extracted from ISF; τ_α: relaxation time of α relaxation; τ_β: relaxation time of β relaxation; $\tau_{\rm m}$: characteristic time of mobile percolation; $\tau_{\rm im}$: characteristic time of immobile percolation.

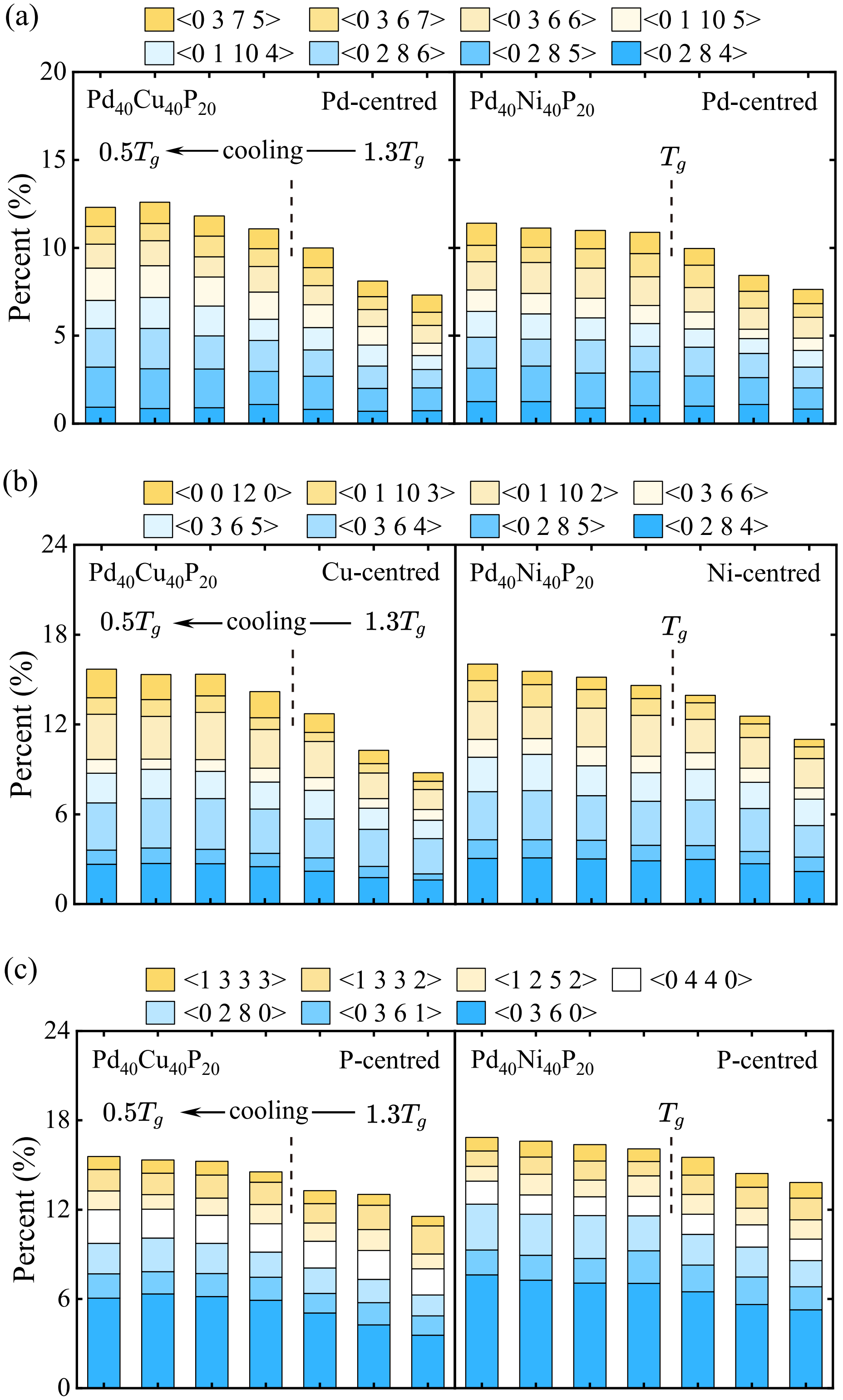

FIG. S26. The evolution of (a) Pd, (b) Cu/Ni and (c) P centered Voronoi polyhedron during continuous cooling in $Pd_{40}Cu_{40}P_{20}$ and $Pd_{40}Ni_{40}P_{20}$ glasses. Except for the P atoms, the other type atoms do not exhibit a dominant voronoi polyhedron.

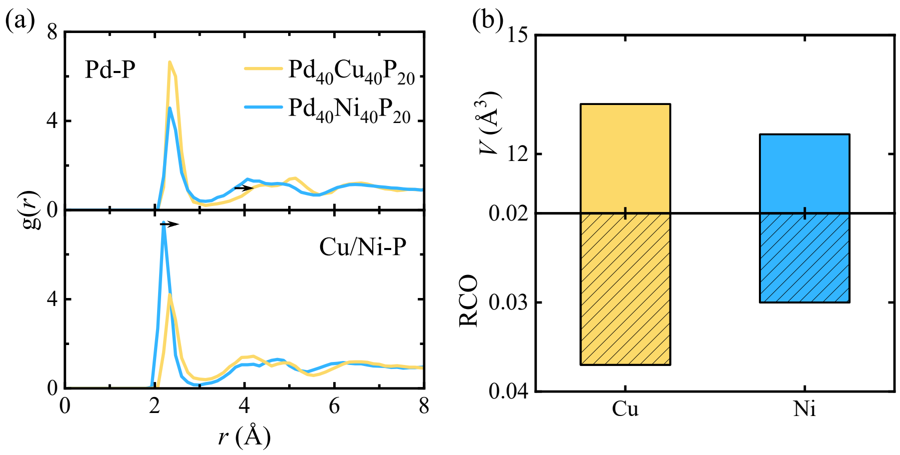

FIG. S27. (a) The partial radial distribution function (Pd-P, upper panel; Cu/Ni-P lower panel) of $Pd_{40}Cu_{40}P_{20}$ and $Pd_{40}Ni_{40}P_{20}$ glasses. The intensity of the Pd-P in $Pd_{40}Cu_{40}P_{20}$ model is greater than that in $Pd_{40}Ni_{40}P_{20}$ model. Conversely, the intensity of Cu-P is significantly lower than that of Ni-P, and the first neighbor distance (the position of the first peak) of Cu-P is greater than that of Ni-P. (b) The average voronoi volume V and the average reduced center offset RCO of Cu/Ni. ROC $= (r_i - r_c)/\sqrt[3]{V}$, where r_i and r_c are the positions of the center atom i and the centroid in a voronoi polyhedron, respectively. Their demonstrate that Cu atoms in $Pd_{40}Cu_{40}P_{20}$ model have larger Voronoi volumes and reduced centroid offset compared to Ni atoms in $Pd_{40}Ni_{40}P_{20}$ model. That is, the Cu atoms with more free space to move in $Pd_{40}Cu_{40}P_{20}$ model.

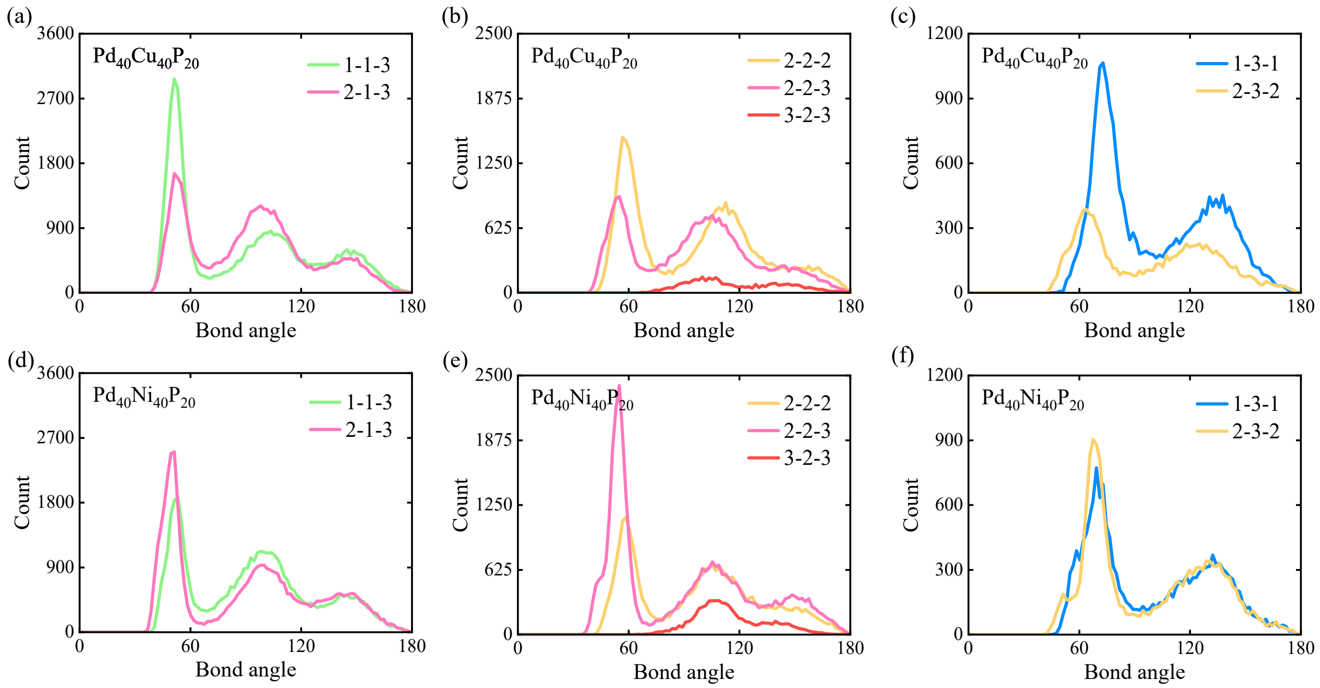

FIG. S28. The bond-angle distributions of $Pd_{40}Cu_{40}P_{20}$ and $Pd_{40}Ni_{40}P_{20}$ glasses. Here, 1 represents Pd atoms, 2 represents Cu/Ni atoms, and 3 represents P atoms. Only distributions with significant differences are included. Two atoms are recognized as bonded if the distance between them is less than 3.6 Å. It is noteworthy that there are almost no P-P bonds, which means that the neighbors of the P atoms consist only of Pd and Cu/Ni atoms.

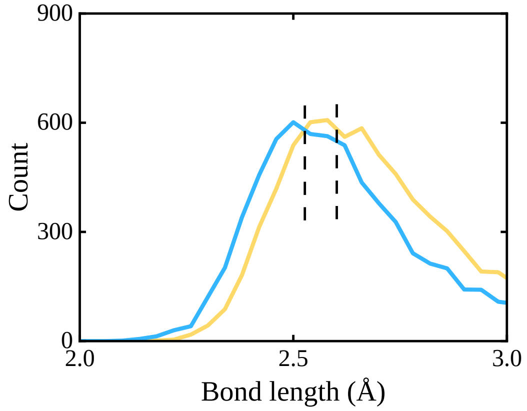

FIG. S29. The bond-length distributions of Cu-P (yellow) and Ni-P (blue) bond in $Pd_{40}Cu_{40}P_{20}$ and $Pd_{40}Ni_{40}P_{20}$ glasses.

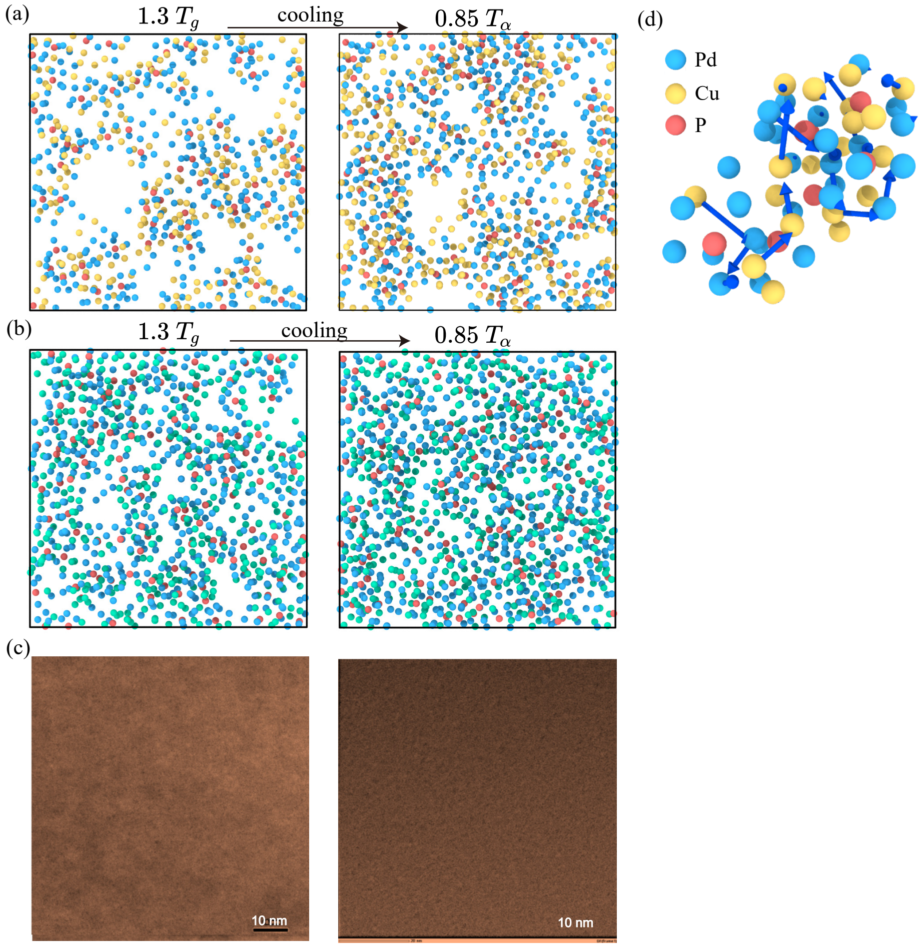

FIG. S30. Solid-like network and string-like motion. (a) 10 Å configuration slice of P-centered <0 3 6 0>, <0 2 8 0>, and <0 4 4 0> polyhedra, as well as their neighbours, in the $Pd_{40}Cu_{40}P_{20}$ (Pd, blue; Cu, yellow; P, red) glass at $1.3T_g$ (left) and $0.85T_\alpha$ (right). (b) show the results of $Pd_{40}Ni_{40}P_{20}$ glass (Pd, blue; Cu, green; P, red), utilizing the same strategy as (a). (c) High-angle annular dark-field micrograph (scale bar: 10 nm) of as-cast $Pd_{40}Cu_{40}P_{20}$ (left) and $Pd_{40}Ni_{40}P_{20}$ (right) glasses. (d) String-like motions in P-centered polyhedral networks, exemplified by $Pd_{40}Cu_{40}P_{20}$ glass.

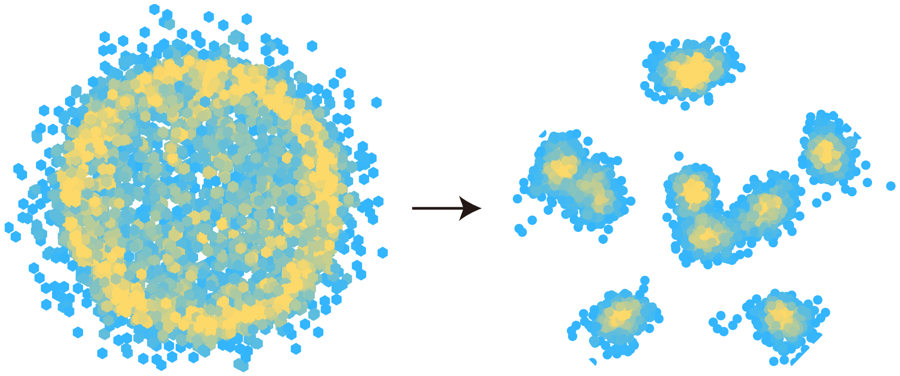

FIG. S31. The Individual Template Cluster Alignment Strategy, exemplified by P-centered <0 3 6 0>polyhedra. The template possesses the smallest RCO in these polyhedra.

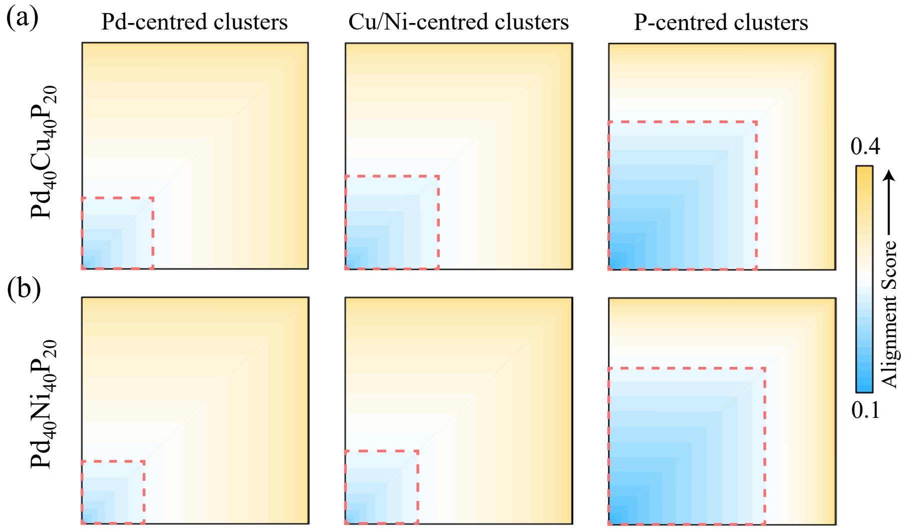

FIG. S32. Alignment score of Pd-centered, Cu/Ni-centered, and P-centered clusters, using the pair-wise cluster alignment strategy. The distribution with higher matching (small score) indicated by more blue; the red dashed line marks the region where the alignment score is less than 0.20. The alignment score quantifies the similarity between two clusters by comparing the positions of their atoms after optimal transformations. During an alignment process, each atom in cluster A is paired with the closest atom in cluster B based on the Euclidean distance between their coordinates. The score for each atom pair i and j is calculated as $\text{score}_{ij} = (r_i - r_j)^2/b_j^2$. Where r_i and r_j are the positions of the atoms of cluster A and cluster B, respectively. And b_j is the bond length of atom in cluster B. The total alignment score is the root mean square of the individual pairwise scores: $\text{Score} = \sqrt{\frac{1}{N-1}\Sigma s_{ij}}$

(a)

$$\mathrm{ASF} = \frac{Vor_{\mathrm{Area\_min}}}{Vor_{\mathrm{Area\_max}}} = \frac{\sum_i^n \min(Vor_{Ai}, Vor_{Bi})}{\sum_i^n \max(Vor_{Ai}, Vor_{Bi})}$$

$$\mathrm{ESF} = \langle \psi_A | \psi_B \rangle = \sum_{ij} \langle Vor_{Ai} | c_{Ai}^* c_{Bj} | Vor_{Bj} \rangle = \sum_i c_{Ai}^* c_{Bi}$$

$$\mathrm{PCC} = \frac{\sum_{i=1}^n (Vor_{Ai} - \overline{Vor_A})(Vor_{Bi} - \overline{Vor_B})}{\sqrt{\sum_{i=1}^n (Vor_{Ai} - \overline{Vor_A})^2}\sqrt{\sum_{i=1}^n (Vor_{Bi} - \overline{Vor_B})^2}}$$

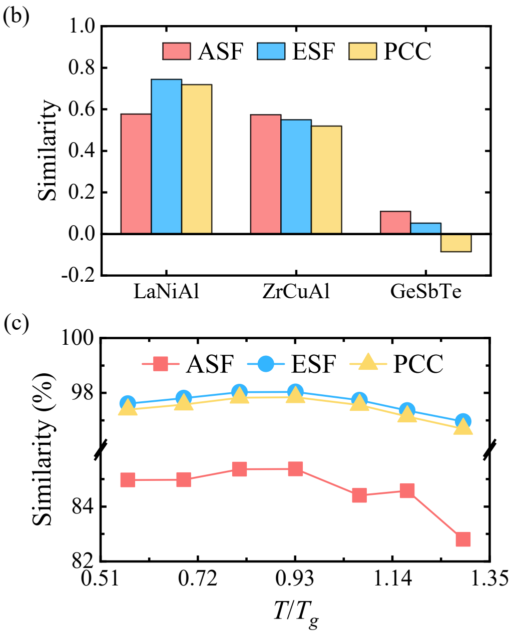

FIG. S33. The structural similarity algorithm based on the Voronoi index. (a) Mathematical expressions for ASF (Area Similarity Function), ESF (Eigenvalue Similarity Function) and PCC (Pearson Correlation Coefficient). (b) Structural similarity between $Pd_{40}Cu_{40}P_{20}$ and other ternary glasses. LaNiAl: $La_{50}Ni_{35}Al_{15}$; ZrCuAl: $Zr_{46}Cu_{46}Al_8$; GeSbTe: $Ge_2Sb_2Te_5$. (c) Structural similarity between $Pd_{40}Cu_{40}P_{20}$ and $Pd_{40}Ni_{40}P_{20}$ glasses during continuing cooling.

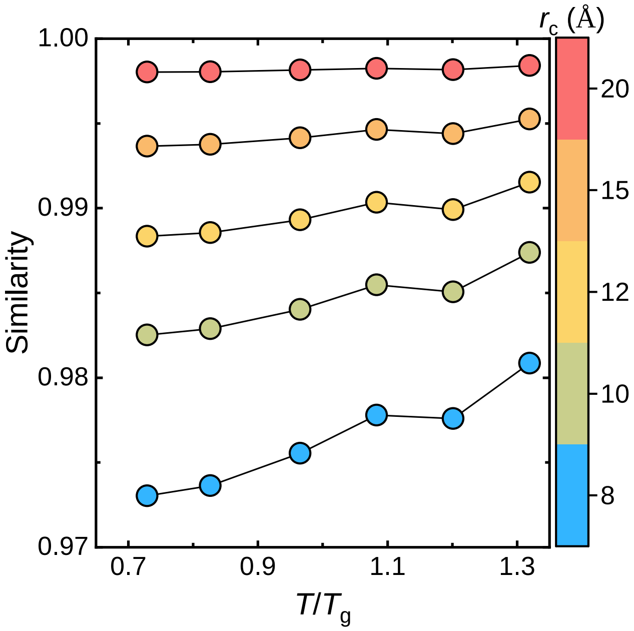

FIG. S34. Similarity between the two models as a function of temperature at different cutoff radius.

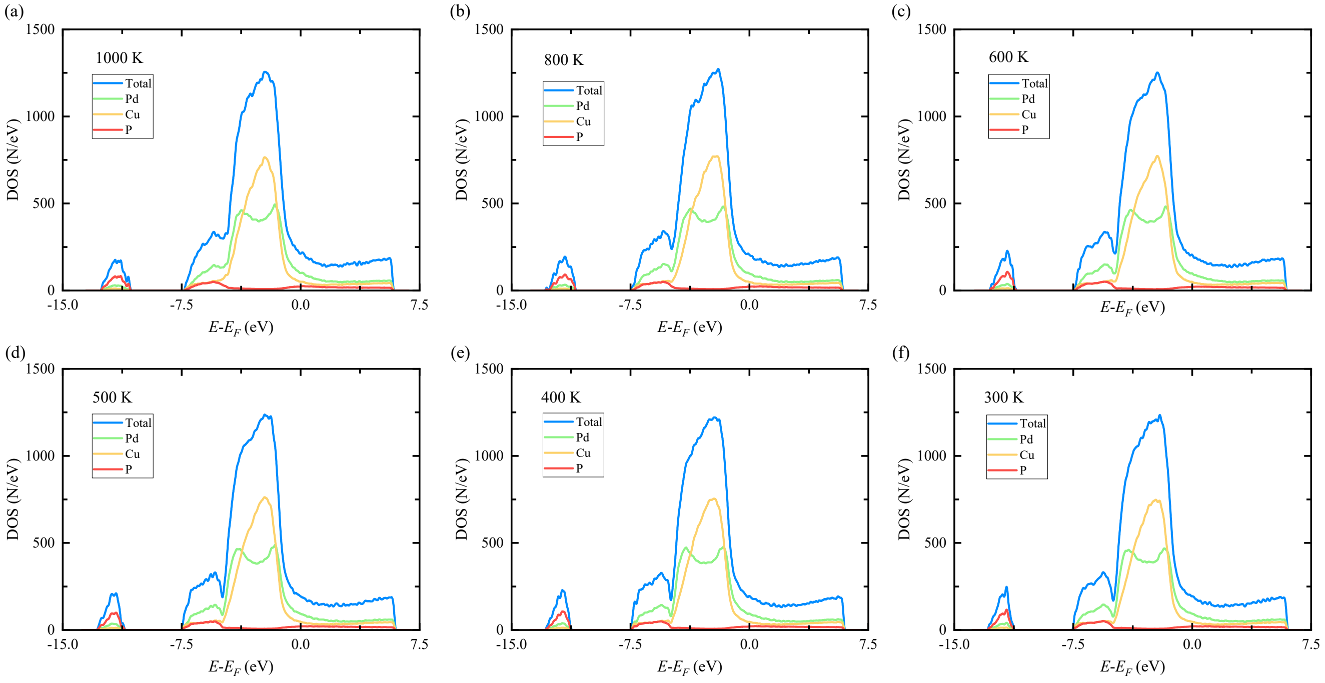

FIG. S35. Density of states (DOS) for a range of temperatures of 500-atom $Pd_{40}Cu_{40}P_{20}$ glass with a cooling rate $R = 2$ K/ns

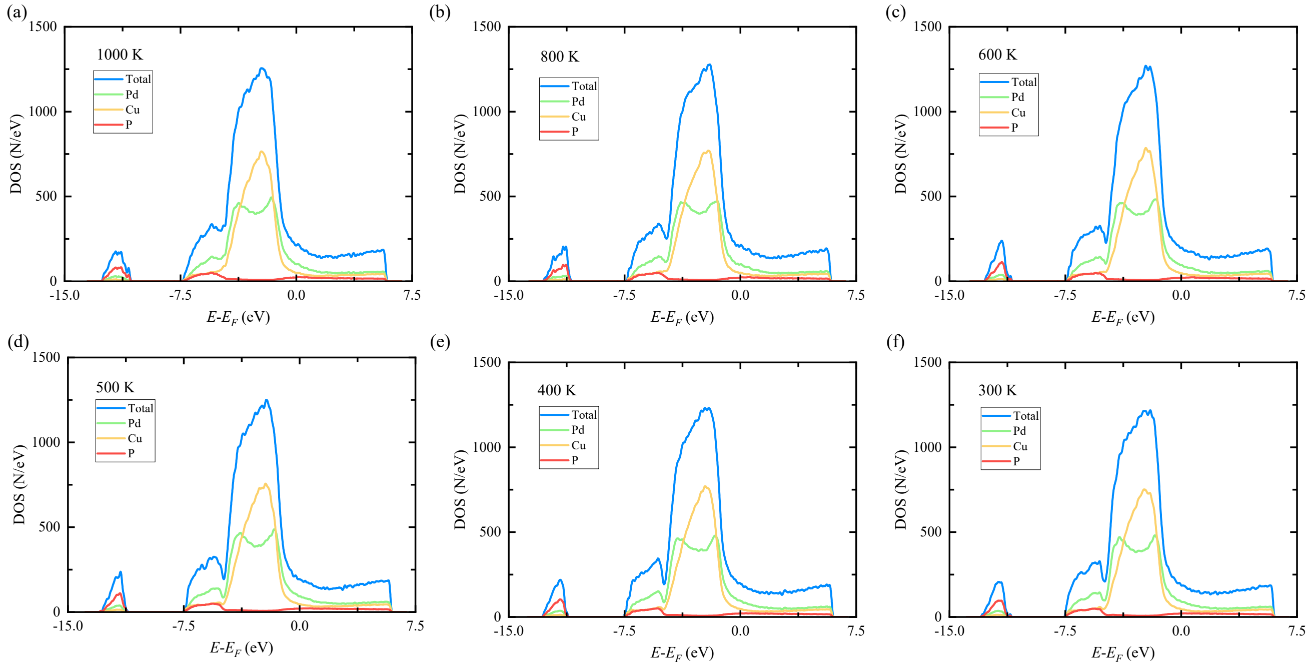

FIG. S36. Density of states (DOS) for a range of temperatures of 500-atom $Pd_{40}Cu_{40}P_{20}$ glass with a cooling rate $R = 10$ K/ns

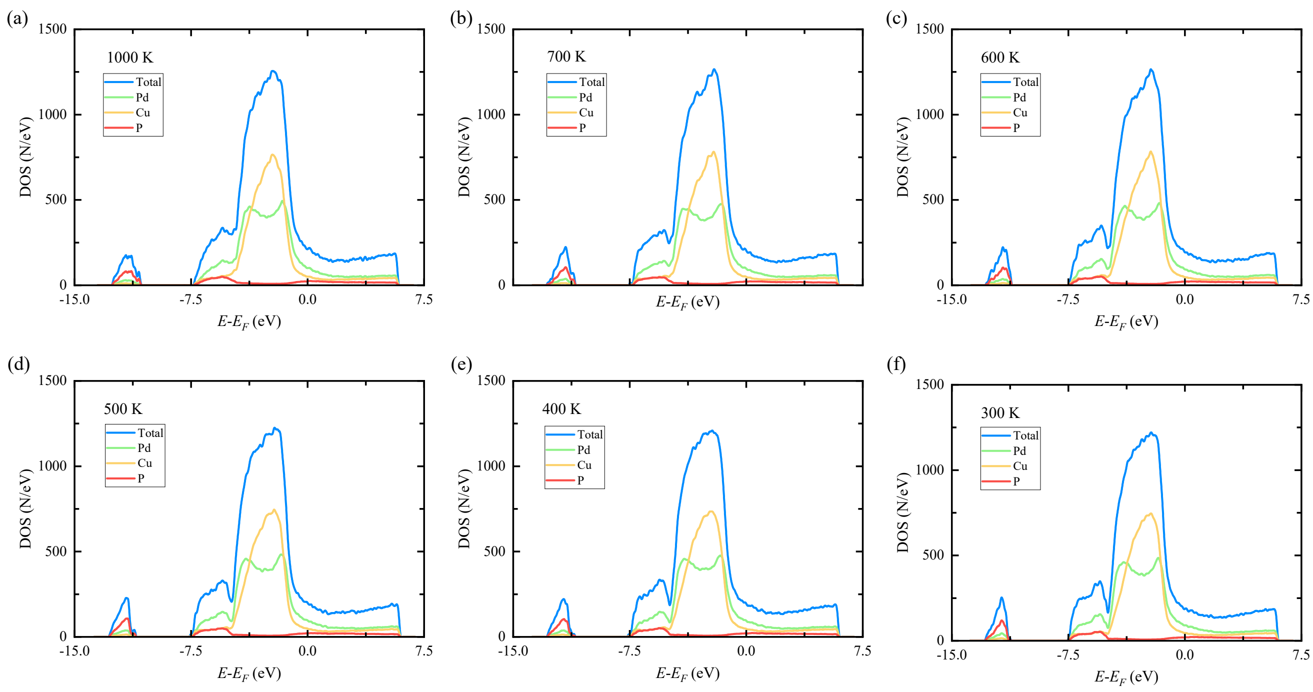

FIG. S37. Density of states (DOS) for a range of temperatures of 500-atom $Pd_{40}Cu_{40}P_{20}$ glass with a cooling rate $R = 100$ K/ns

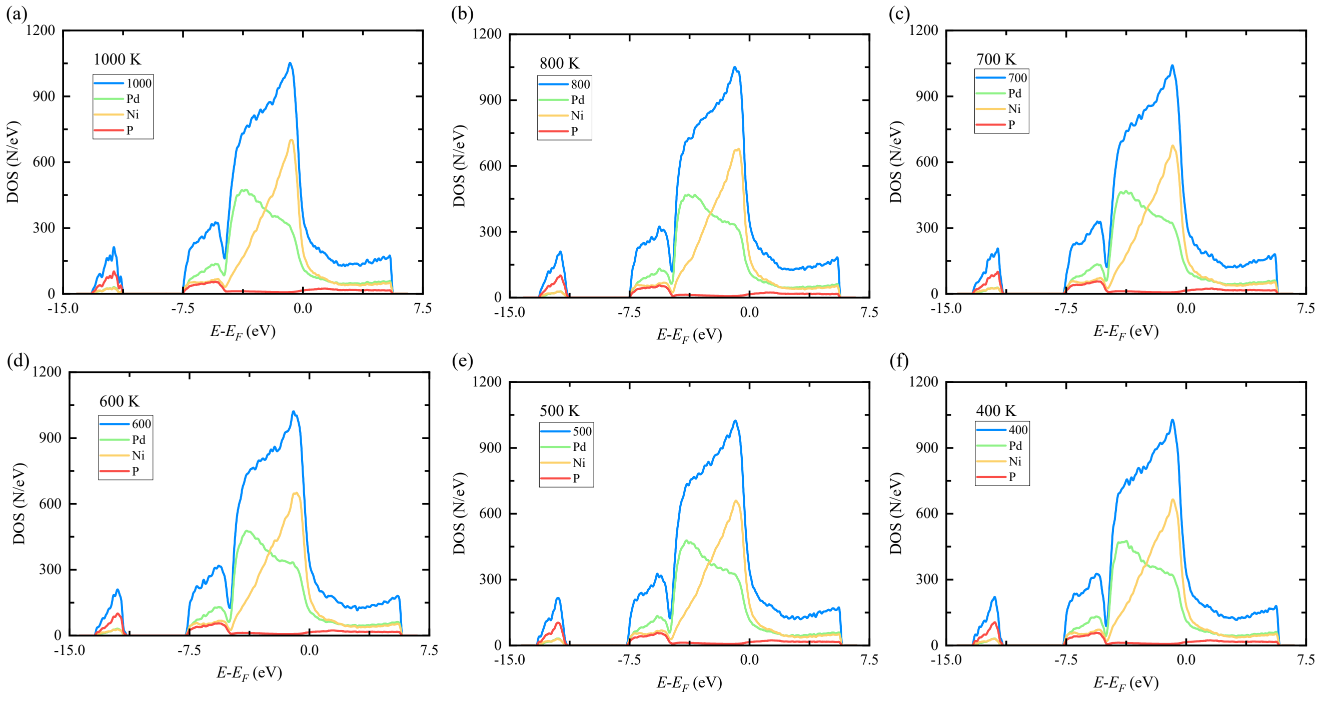

FIG. S38. Density of states (DOS) for a range of temperatures of 500-atom $Pd_{40}Ni_{40}P_{20}$ glass with a cooling rate $R = 2$ K/ns

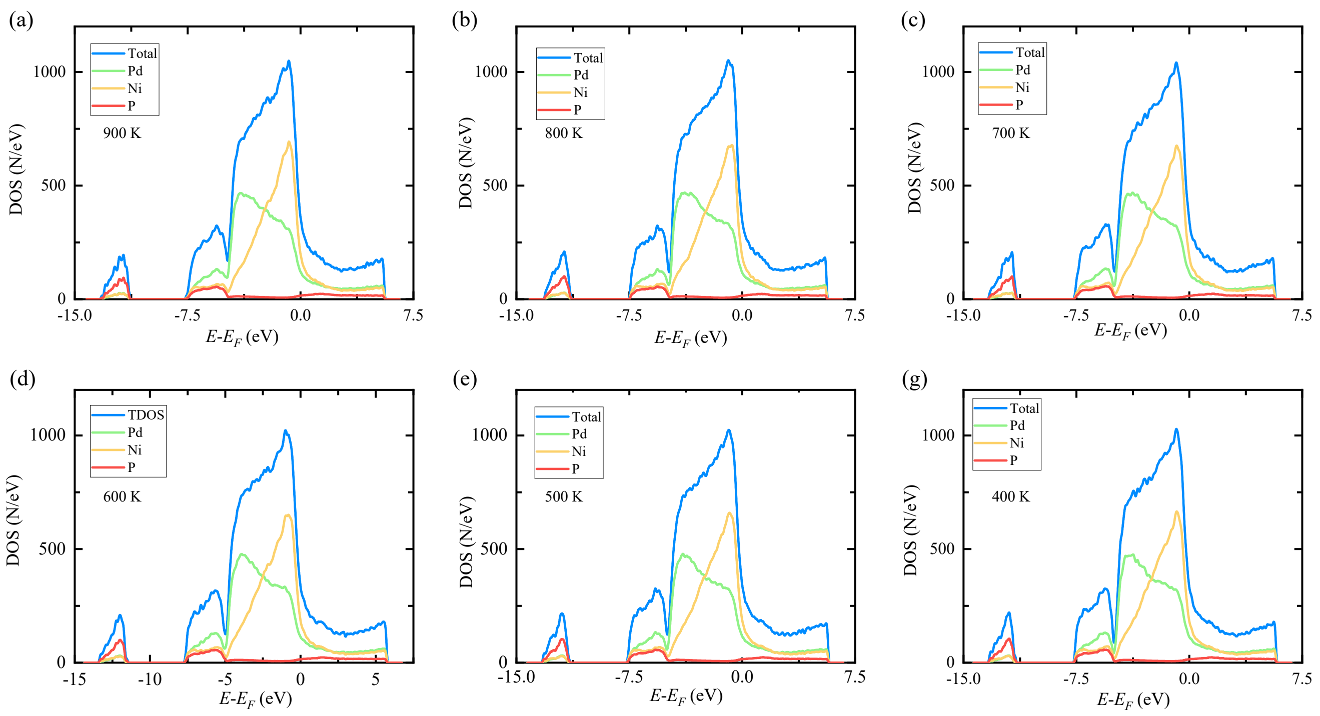

FIG. S39. Density of states (DOS) for a range of temperatures of 500-atom $Pd_{40}Ni_{40}P_{20}$ glass with a cooling rate R = 10 K/ns

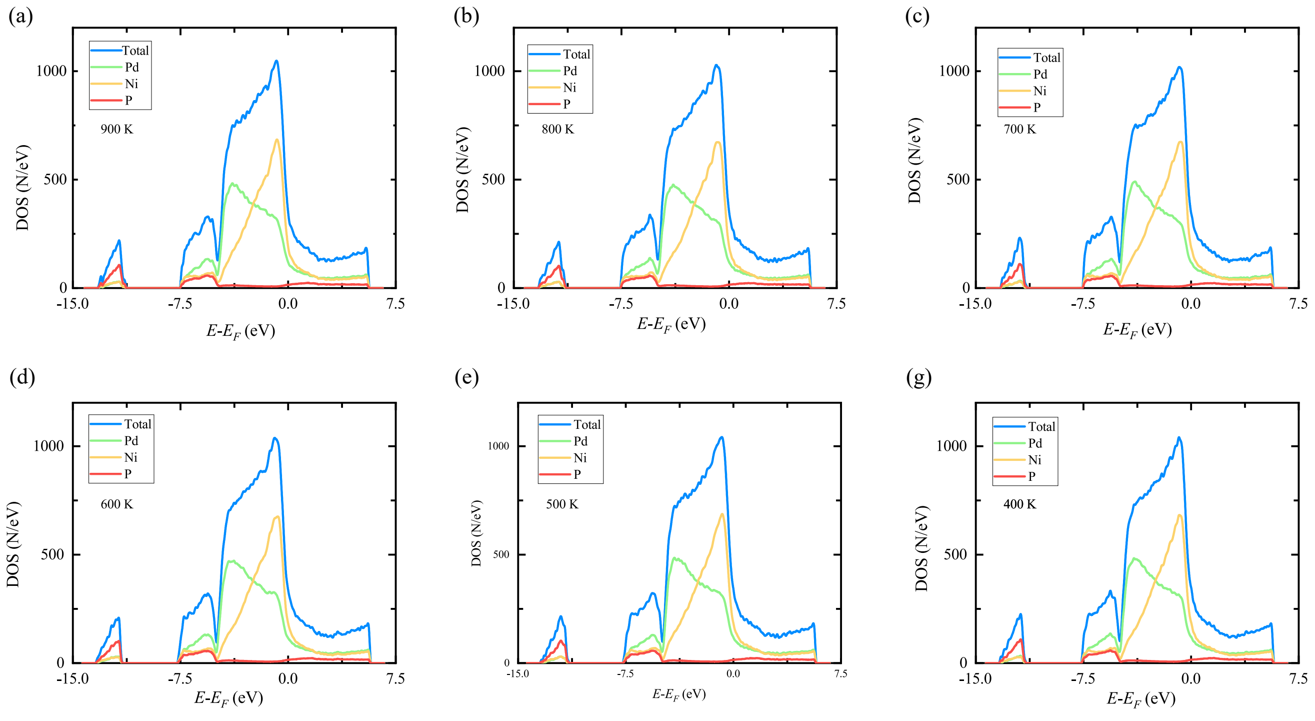

FIG. S40. Density of states (DOS) for a range of temperatures of 500-atom $Pd_{40}Ni_{40}P_{20}$ glass with a cooling rate R = 100 K/ns

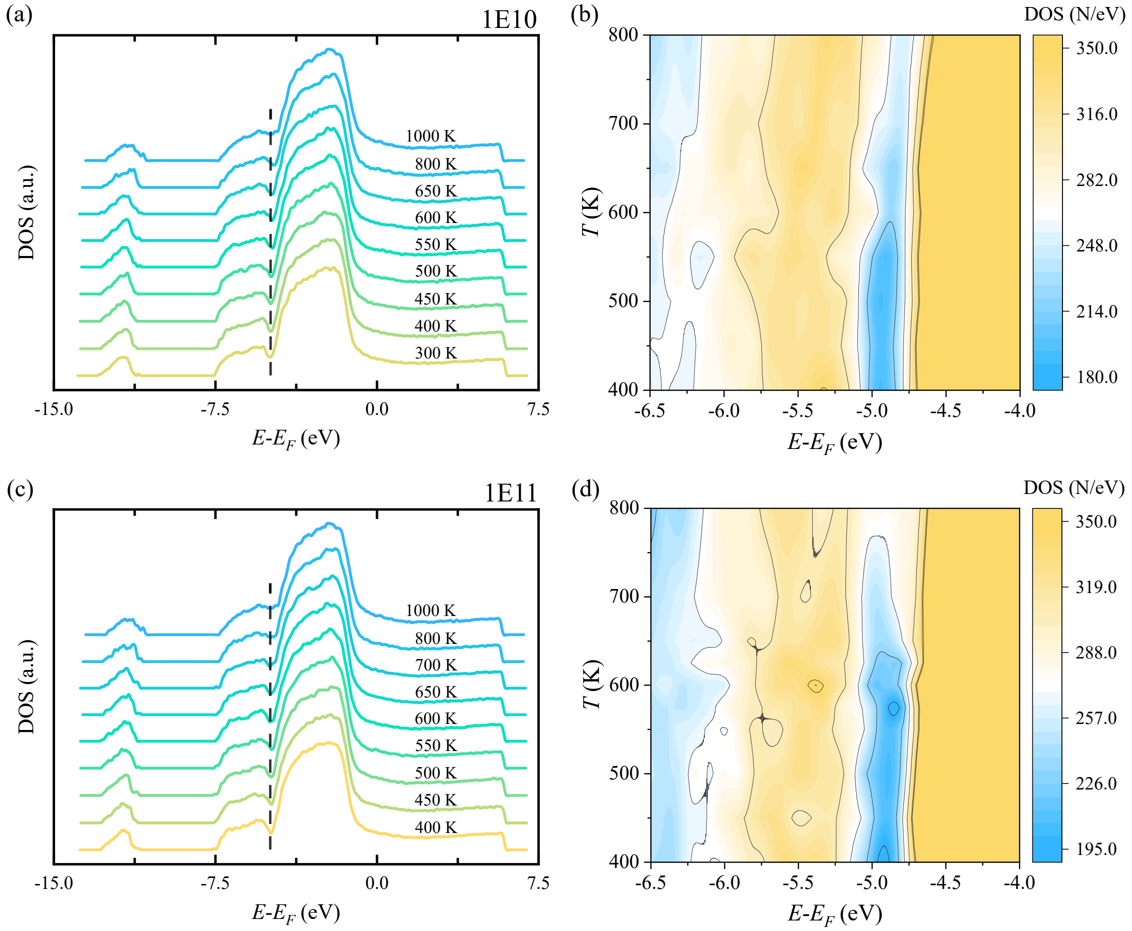

FIG. S41. Contour map of the total density of states depicting the pseudo-energy gap for $Pd_{40}Cu_{40}P_{20}$ glass with cooling rate $R = 10$ (a-b) and 100 (c-d) K/ns.

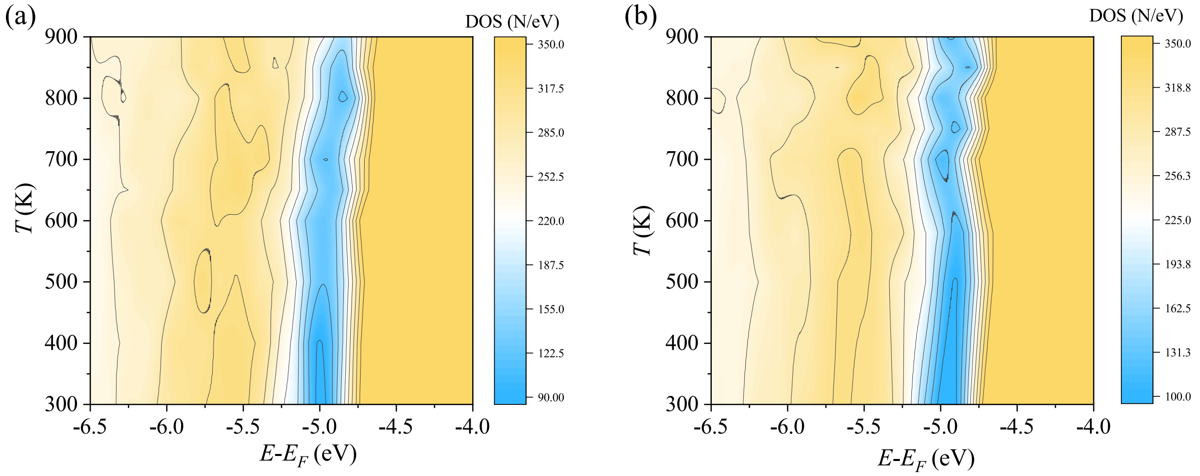

FIG. S42. Contour map of the total density of states depicting the pseudo-energy gap for $Pd_{40}Ni_{40}P_{20}$ glass with cooling rate $R = 10$ (a) and 100 (b) K/ns.

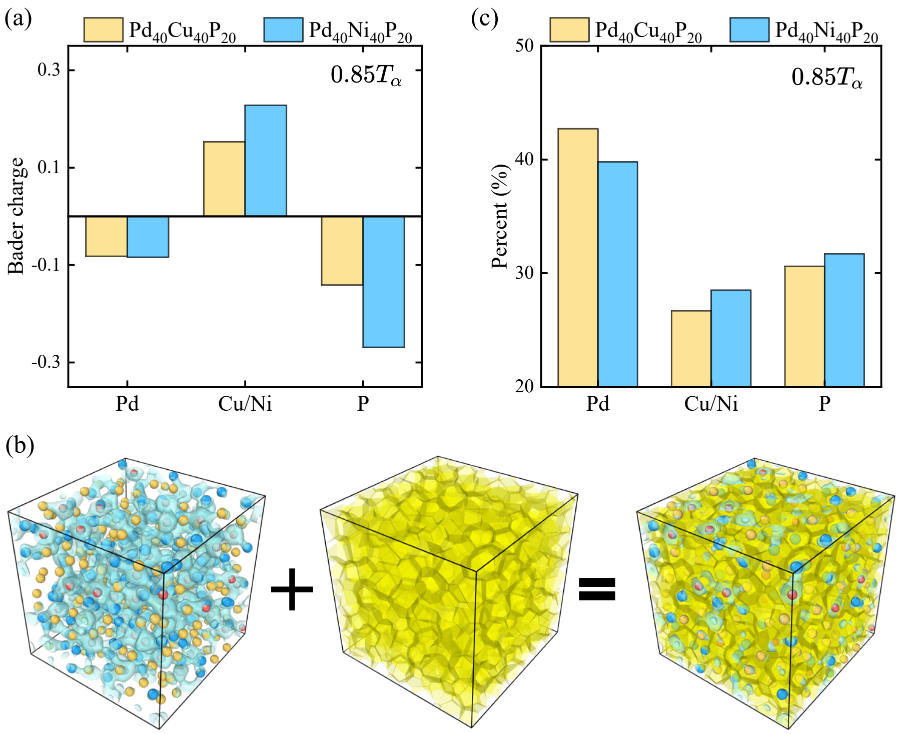

FIG. S43. (a) Bader charge analysis of $Pd_{40}Cu_{40}P_{20}$ and $Pd_{40}Ni_{40}P_{20}$ glasses at $0.85T_\alpha$. (b) A schematic diagram of electronic assignment of the "Hybrid" region based on bader partitioning. (c) Electronic assignment of the "Hybrid" region of both glasses at $0.85T_\alpha$.

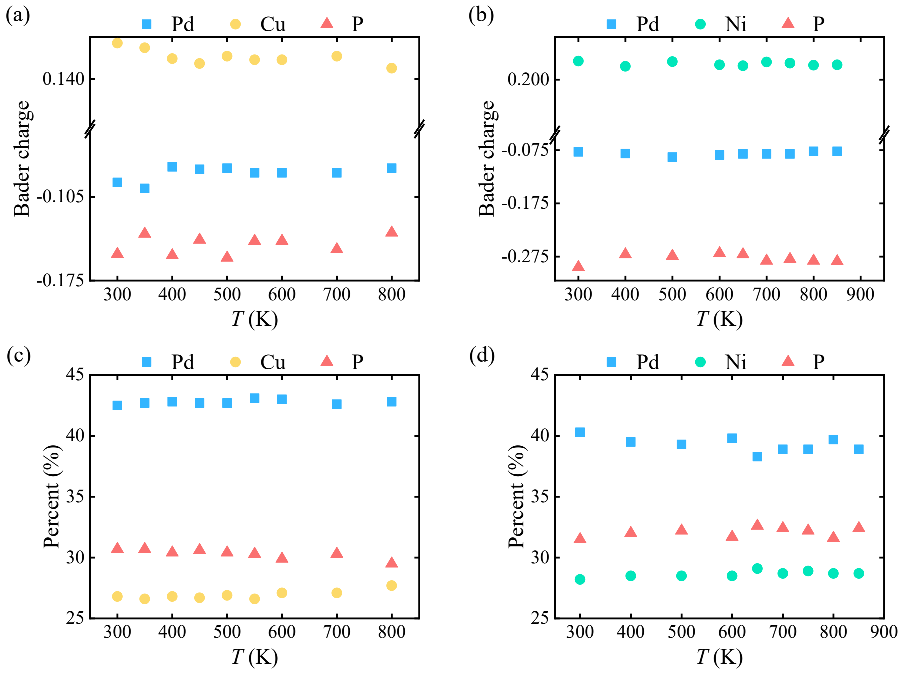

FIG. S44. Bader charge analysis of (a) $Pd_{40}Cu_{40}P_{20}$ and (b) $Pd_{40}Ni_{40}P_{20}$ glasses during continuous cooling. (c-d) Electronic assignment of the "Hybrid" region of both glasses during continuous cooling.

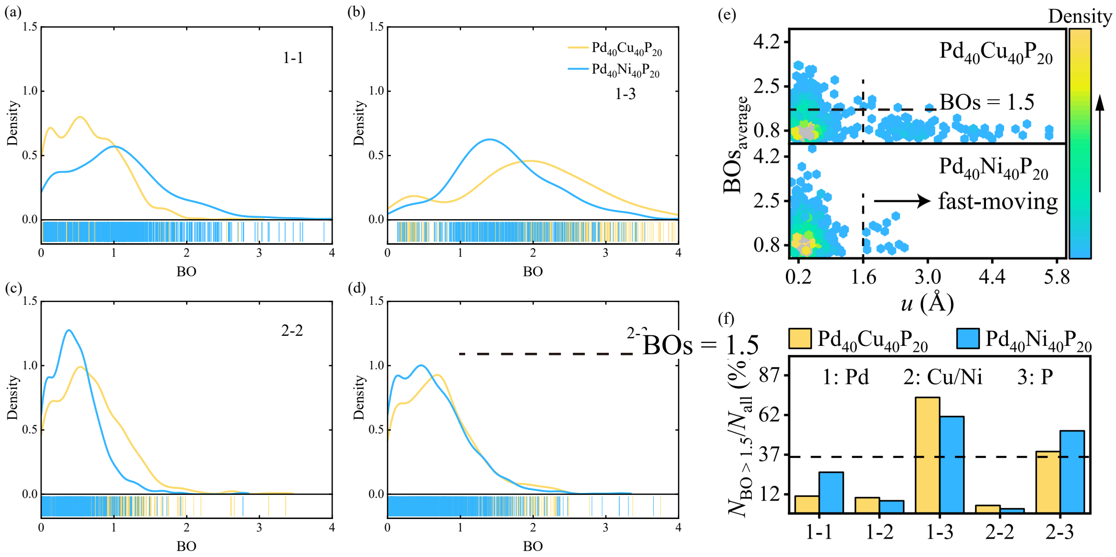

FIG. S45. The bond order distributions of (a) Pd-Pd, (b) Pd-P, (c) Cu/Ni-Cu/Ni, and (d) Cu/Ni-P bonds in $Pd_{40}Cu_{40}P_{20}$ and $Pd_{40}Ni_{40}P_{20}$ glasses. Consider only the nearest neighboring atoms. The P-P bond is almost non-existent in both glasses. (e) Correlation between the averaged BO ($BO_{average}$) and atomic displacement (u) in $Pd_{40}Cu_{40}P_{20}$ (upper panel) and $Pd_{40}Ni_{40}P_{20}$ (lower panel) glasses at $0.85T_{\alpha}$. The dashed line separates fast-moving atoms from nearly stationary ones. (f) Percentage of strong bonds (BO > 1.5) among all bonds in $Pd_{40}Cu_{40}P_{20}$ (yellow) and $Pd_{40}Ni_{40}P_{20}$ (blue) glasses at $0.85T_{\alpha}$. Here, 1 represents Pd atoms, 2 represents Cu/Ni atoms, and 3 represents P atoms.

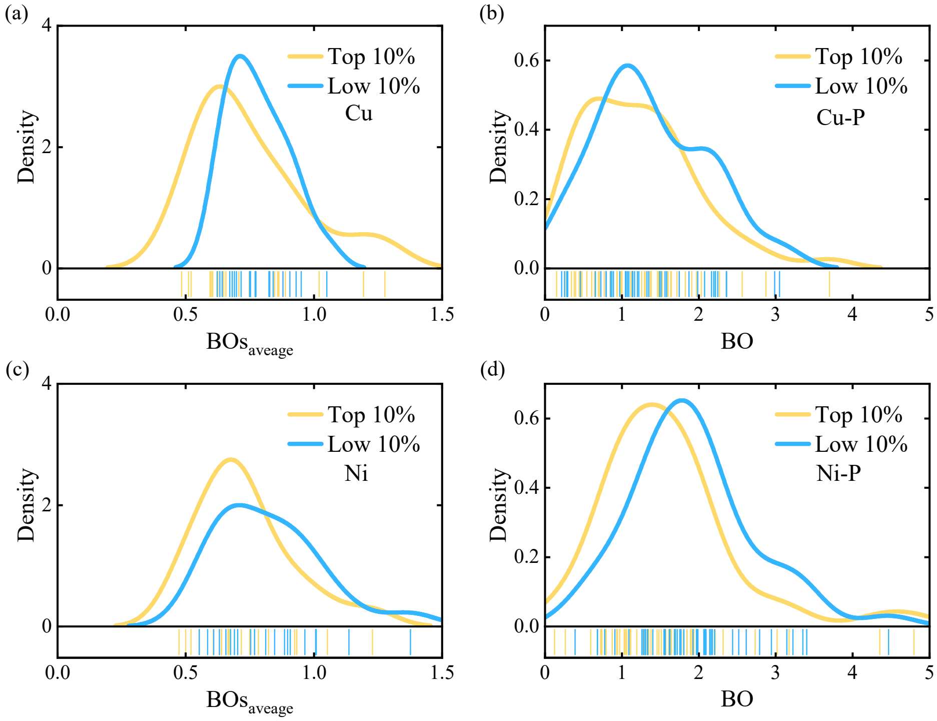

FIG. S46. (a,c) Average bond orders distributions of the 10% largest and 10% smallest displacements in Cu/Ni atoms. (b,d) Cu/Ni-P bond orders distributions of the 10% largest and 10% smallest displacements in Cu/Ni atoms. This suggests that the weak bond orders result in a high tendency for Cu to move.

[1] A. Stukowski, Visualization and analysis of atomistic simulation data with ovito–the open visualization tool, Modelling and Simulation in Materials Science and Engineering **18**, 015012 (2009).

[2] G. Kresse and J. Hafner, Ab initio molecular dynamics for liquid metals, Physical Review B **47**, 558 (1993).

[3] L. Zhang, J. Han, H. Wang, W. A. Saidi, R. Car, and E. Weinan, End-to-end symmetry preserving inter-atomic potential energy model for finite and extended systems, in *Proceedings of the 32nd International Conference on Neural Information Processing Systems*, NIPS'18 (Curran Associates Inc., Red Hook, NY, USA, 2018) p. 4441–4451.

[4] H. Wang, L. Zhang, J. Han, and W. E, Deepmd-kit: A deep learning package for many-body potential energy representation and molecular dynamics, Computer Physics Communications **228**, 178 (2018).

[5] D. Lu, H. Wang, M. Chen, L. Lin, R. Car, W. E, W. Jia, and L. Zhang, 86 pflops deep potential molecular dynamics simulation of 100 million atoms with ab initio accuracy, Computer Physics Communications **259**, 107624 (2021).

[6] J. Zeng, D. Zhang, D. Lu, P. Mo, Z. Li, Y. Chen, M. Rynik, L. Huang, Z. Li, S. Shi, Y. Wang, H. Ye, P. Tuo, J. Yang, Y. Ding, Y. Li, D. Tisi, Q. Zeng, H. Bao, Y. Xia, J. Huang, K. Muraoka, Y. Wang, J. Chang, F. Yuan, S. L. Bore, C. Cai, Y. Lin, B. Wang, J. Xu, J.-X. Zhu, C. Luo, Y. Zhang, R. E. A. Goodall, W. Liang, A. K. Singh, S. Yao, J. Zhang, R. Wentzcovitch, J. Han, J. Liu, W. Jia, D. M. York, W. E, R. Car, L. Zhang, and H. Wang, DeePMD-kit v2: A software package for deep potential models, The Journal of Chemical Physics **159**, 054801 (2023).

[7] L. Gao, H.-B. Yu, T. B. Schrøder, and J. C. Dyre, Unified percolation scenario for the α and β processes in simple glass formers, Nature Physics **21**, 471 (2025).

[8] H.-R. Zhang, L. Gao, Y.-H. Ye, J.-X. Zhang, T. Zhang, Q.-Z. Bu, Q. Yang, Z.-W. Zhu, S. Wei, and H.-B. Yu, Fragility crossover mediated by covalent-like electronic interactions in metallic liquids, Materials Futures **3**, 025002 (2024).
